# Characterizing Spatial Associations Between GluCEST MRI and Neurotransmitter Receptor Density in the Human Cortex

**DOI:** 10.1002/hbm.70442

**Published:** 2025-12-23

**Authors:** Maggie K. Pecsok, Golia Shafiei, Ally Atkins, Monica E. Calkins, Ruben C. Gur, Ravi Prakash Reddy Nanga, Ravinder Reddy, Melanie A. Matyi, Jacquelyn Stifelman, Heather Robinson, Erica B. Baller, Russell T. Shinohara, Kosha Ruparel, Kristin A. Linn, Daniel H. Wolf, Theodore D. Satterthwaite, Corey T. McMillan, David Roalf

**Affiliations:** ^1^ Brain Behavior Lab, Neurodevelopment and Psychosis Section, Penn‐Chop Lifespan Brain Institute, Department of Psychiatry University of Pennsylvania Perelman School of Medicine Philadelphia Pennsylvania USA; ^2^ Penn Lifespan Informatics & Neuroimaging Center, Lifespan Brain Institute, Department of Psychiatry University of Pennsylvania Perelman School of Medicine Philadelphia Pennsylvania USA; ^3^ Center for Advanced Metabolic Imaging in Precision Medicine (CAMIPM), Perelman School of Medicine, University of Pennsylvania Philadelphia Pennsylvania USA; ^4^ Department of Neurology University of Pennsylvania Perelman School of Medicine Philadelphia Pennsylvania USA; ^5^ Penn Statistics in Imaging and Visualization Endeavor (PennSIVE) Perelman School of Medicine, University of Pennsylvania Philadelphia Pennsylvania USA

## Abstract

Glutamate‐weighted Chemical Exchange Saturation Transfer (GluCEST) captures in vivo glutamate (Glu) levels with high spatial resolution and has been used to assess glutamatergic function in healthy and clinical populations. While GluCEST is well‐validated against proton magnetic resonance spectroscopy (^1^H‐MRS), its correspondence with local expression of glutamatergic neurotransmitter receptors remains unclear. Recent initiatives, such as Neuromaps, have collated positron emission tomography (PET) data into curated, publicly available databases, providing a novel opportunity to establish convergence in the regional distribution of GluCEST and normative receptor density maps. Here, we examine the spatial correspondence between GluCEST signal and PET‐based cortical receptor density levels of N‐methyl‐D‐aspartate (NMDA), metabotropic glutamate receptor 5 (mGluR5), and gamma‐aminobutyric acid A (GABA_A_). A cohort of 86 participants (age: 22.7 years [3.7 years], 45% female) included 34 individuals with no psychiatric history, 31 participants with significant sub‐threshold psychosis symptoms, and 21 participants with first‐episode psychosis. All participants underwent 7T GluCEST imaging. Data were processed using in‐house and field‐standard pipelines. Mean receptor density levels were computed using the Neuromaps PET receptor density data. GluCEST and Neuromaps data were parcellated using the Cammoun 500 atlas. Pearson correlations assessed the correspondence between GluCEST signal and PET‐based receptor density, and spin tests were used for empirical significance testing of the spatial correlations across all parcels. Sensitivity analyses examined the effect of age, sex, and diagnosis and other covariates. Exploratory analyses assessed regional variability across cytoarchitecturally defined von Economo regions and overall trends with gene expression. Analyses were performed in Python and R. GluCEST signal converged with the regional distribution of both NMDA (*r* = 0.23, p_spin_ = 0.039) and GABA_A_ (*r* = 0.35, p_spin_ = 0.004). There was no significant effect for mGluR5 (*r* = 0.09, p_spin_ > 0.05). Exploratory analyses indicated that cytoarchitecturally defined von Economo regions showed variable GluCEST‐receptor association patterns across the cortex and that gene expression patterns generally correspond with receptor density findings. Our findings reveal a positive spatial association between GluCEST signal in a transdiagnostic cohort and atlas‐based PET‐derived cortical receptor density of NMDA and GABA_A_, and a nominal positive association with mGluR5. The association between GluCEST and NMDA suggests that regions with dense ionotropic Glu receptors exhibit higher Glu levels, while the coupling between GluCEST and GABA_A_ may reflect tight regulation of excitation‐inhibition balance. Regional differences in these associations point to the potential influence of local cytoarchitectural specialization on Glu‐receptor dynamics. These results advance our understanding of the neurobiological basis of GluCEST and highlight its potential utility as a non‐invasive tool for probing receptor‐mediated glutamatergic neurotransmission.

## Graphical abstract

We leveraged in vivo 7T GluCEST MRI and normative PET receptor maps to examine the spatial covariation between cortical glutamate levels and receptor expression. GluCEST signal showed positive spatial associations with NMDA and GABA_A_, but not mGluR5. These findings enhance our understanding of GluCEST and glutamate‐receptor dynamics across the cortex.

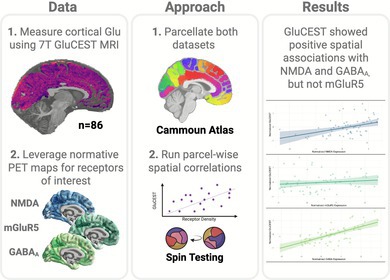

## 1 Introduction

Ultra‐high field 7T Glutamate‐weighted Chemical Exchange Saturation Transfer (GluCEST) is a valuable non‐invasive magnetic resonance imaging (MRI) technique that measures in vivo Glu levels with unprecedented spatial resolution and repeatability (Cai et al. 2012; Nanga et al. 2018). Thus, GluCEST has emerged as a powerful tool for investigating glutamatergic function and dysfunction in a wide variety of disorders (Cember et al. 2022; Davis et al. 2015; Jia et al. 2020; Roalf et al. 2017). While the technique has been well‐validated against proton magnetic resonance spectroscopy (^1^H‐MRS) (Bagga et al. 2018; Schmitz‐Abecassis et al. 2024; Sydnor et al. 2021), it remains unclear how the spatial distribution of GluCEST‐measured Glu is associated with the expression of glutamatergic and GABAergic receptors in the brain. By analyzing how Glu levels co‐vary with receptor densities in the present study, we aim to anchor GluCEST measurements in a neurobiological framework that contextualizes both basic and translational findings and enhances understanding of the interplay between Glu and its associated receptor systems across the cortex.

Emerging evidence suggests that Glu may modulate the local expression and function of key excitatory and inhibitory receptors in the brain, including N‐methyl‐D‐aspartate (NMDA), metabotropic glutamate receptor 5 (mGluR5), and gamma‐aminobutyric acid A (GABA_A_). For example, Glu binding to the GluN2B subunit of NMDA receptors has been shown to regulate surface trafficking (She et al. 2012), while synaptic Glu levels modulate NMDA receptor activity in inhibitory neurons (Yao et al. 2018). Glu levels have also been shown to influence the spatiotemporal dynamics of mGluR5 trafficking in astrocytes (Lee and Parpura 2016). Additionally, Glu may indirectly regulate the expression of GABA_A_ receptor subunits through crosstalk between GABA_A_ and NMDA receptor density (Memo et al. 1991). At the transcriptional level, these receptors are encoded by distinct gene families (GRIN for NMDA, GRM for mGluR5, and GABR for GABA_A_) (Chou et al. 2022; Daggett et al. 1995; Yashiro and Philpot 2008), though how Glu levels may affect the transcription and subsequent expression of these genes has not been fully elucidated. Notably, these receptors exhibit functional heterogeneity across cortical regions with distinct cytoarchitectural properties. For example, mGluR5 and NMDA play different roles in synaptic plasticity mechanisms in the hippocampus compared to the primary visual cortex (Chokshi et al. 2019; Sidorov et al. 2015). There is also evidence of functional heterogeneity of GABA_A_ across cortical regions (Chen et al. 2023; Schwarzer et al. 2001). These in vitro findings suggest a potential link between local Glu levels and receptor density, which may vary across different brain regions.

Recent studies have explored the relationship between in vivo neurotransmitter levels and receptor density in the human brain using ^1^H‐MRS and positron emission tomography (PET) imaging. While this multimodal approach has shown some associations between PET and spectroscopy measures, findings are mixed and remain methodologically constrained. Critically, no prior work has explicitly tested spatial correlations between in vivo neurotransmitter levels and receptor density, which requires sampling multiple ROIs per subject. Rather, most studies have reported region‐specific PET‐MRS associations. For example, a negative association between GABA_A_ levels (^1^H‐MRS) and GABA_A_ receptor density ([^11^C]‐flumazenil PET) was observed in the frontal eye fields, but not the occipital cortex, of patients with neurofibromatosis type 1 (Violante et al. 2016). In healthy individuals, baseline mGluR5 density ([^18^F]PSS232 PET) was positively correlated with frontal cortex glutamate + glutamine (Glx; ^1^H‐MRS) increases following n‐acetylcysteine administration. Conversely, several studies examining substance use and stress have reported no significant association between mGluR5 density ([^18^F]‐FPEB PET) and Glu or Glx (^1^H‐MRS) in the frontal cortex or striatum of rats and humans (Bdair et al. 2024; De Laat et al. 2018; Martinez et al. 2014). Notably, Beck et al. (2022) used simultaneous PET‐MRS to identify a negative association between hippocampal NMDA receptor density and striatal Glu in patients with psychosis, a population in which Glu abnormalities have been reported (Merritt et al. 2023; Roalf et al. 2017). However, this study did not characterize a direct spatial correlation between these measures.

While prior work provides a valuable conceptual foundation for the current investigation, the studies face a common set of limitations. ^1^H‐MRS is limited by low spatial resolution and spectral overlap between Glu and glutamine (Gln), complicating precise neurotransmitter quantification, particularly at lower field strengths (Yang et al. 2008). Furthermore, most investigations focus on isolated, non‐overlapping regions of interest (ROIs)—such as the frontal cortex or striatum—precluding systematic synthesis of findings. Sampling from multiple regions is necessary to compute spatial correlations between measures; it is also important to sample widely to account for cytoarchitectural heterogeneity across the brain. For example, von Economo regions exhibit distinct laminar organization and computational priorities (Cauda et al. 2013) and may show divergent receptor‐neurotransmitter relationships due to local functional specializations. Hence, there is a critical need to conduct a robust exploration of spatial correlations between neurotransmitter dynamics and receptor density at a whole‐brain scale.

In this study, we leveraged 7T GluCEST and normative PET‐derived receptor density maps to investigate the spatial covariance between GluCEST and NMDA, mGluR5, and GABA_A_ receptor density across the cortex. Compared to its alternatives, 7T GluCEST offers superior spatial resolution and sensitivity, enabling precise Glu quantification and robust assessment of spatial correlations. These advantages also allowed us to explore how Glu‐receptor relationships differ across cytoarchitecturally defined von Economo regions, which is important because these regions reflect fundamental divisions in cortical organization based on distinct laminar patterns, connectivity profiles, and functional specialization (von Economo and Koskinas 1925; Scholtens et al. 2018). In addition, we explored transcriptional data from the Allen Human Brain Atlas (AHBA) to complement PET receptor data, focusing on genes encoding receptor subunits for NMDA, mGluR5, and GABA_A_, as well as enzymes critical for glutamate synthesis and metabolism. We hypothesized that GluCEST would exhibit a positive spatial association with the density of NMDA, mGluR5, and GABA_A_ receptors, reflecting the potentially tight coupling between neurotransmitter availability and receptor density required for functional synaptic signaling. We expected these associations to be robust across cortical regions and regardless of participants' diagnostic status. Lastly, we expected to find directionally similar trends between GluCEST and AHBA‐derived gene expression data. By examining the spatial correspondence between GluCEST and receptor density, this study aims to advance our understanding of GluCEST imaging and glutamatergic signaling in the brain. Such information can enhance the interpretability of GluCEST data in both basic and translational research, paving the way for further elucidating glutamate's role in brain health and disease.

## 2 Methods

### 2.1 Participants

The GluCEST data were collected at the University of Pennsylvania in Philadelphia, Pennsylvania between March 2021 and August 2024. The sample (*N* = 86, age 14–30, 45% female) included 34 participants with no history of significant psychiatric disorders or subthreshold psychosis symptomatology and no history of psychotropic medication use, 31 participants who endorsed significant subthreshold psychosis symptoms, and 21 participants with a diagnosed psychosis spectrum disorder (e.g., Schizophrenia, psychosis NOS, etc.). All participants were either recruited from the local community and online participant databases or through a collaboration with the Penn‐CHOP Lifespan Brain Institute. Assessments were performed by trained and certified assessors (Calkins et al. 2017). All participants were administered the Structured Interview for Prodromal Syndromes (v. 4.0 or higher), a standardized clinical interview through which the severity of positive, negative, and disorganized psychosis symptoms are rated on the Scale of Prodromal Symptoms (SOPS) (Miller et al. 2003). Clinical diagnoses and final SOPS ratings were assigned by consensus conference and review including at least one doctoral level clinician. The Institutional Review Boards of the University of Pennsylvania and the Children's Hospital of Philadelphia (CHOP) approved all procedures. Participants over 18 provided written informed consent, while younger participants gave informed assent with written parental consent. A subset of participants (*n* = 53) completed a follow‐up 7T GluCEST scan session within one year of the initial visit. These data were used as a replication dataset for sensitivity analyses (Table S1).

### 2.2 7T GluCEST MRI Acquisition

**FIGURE 1:**
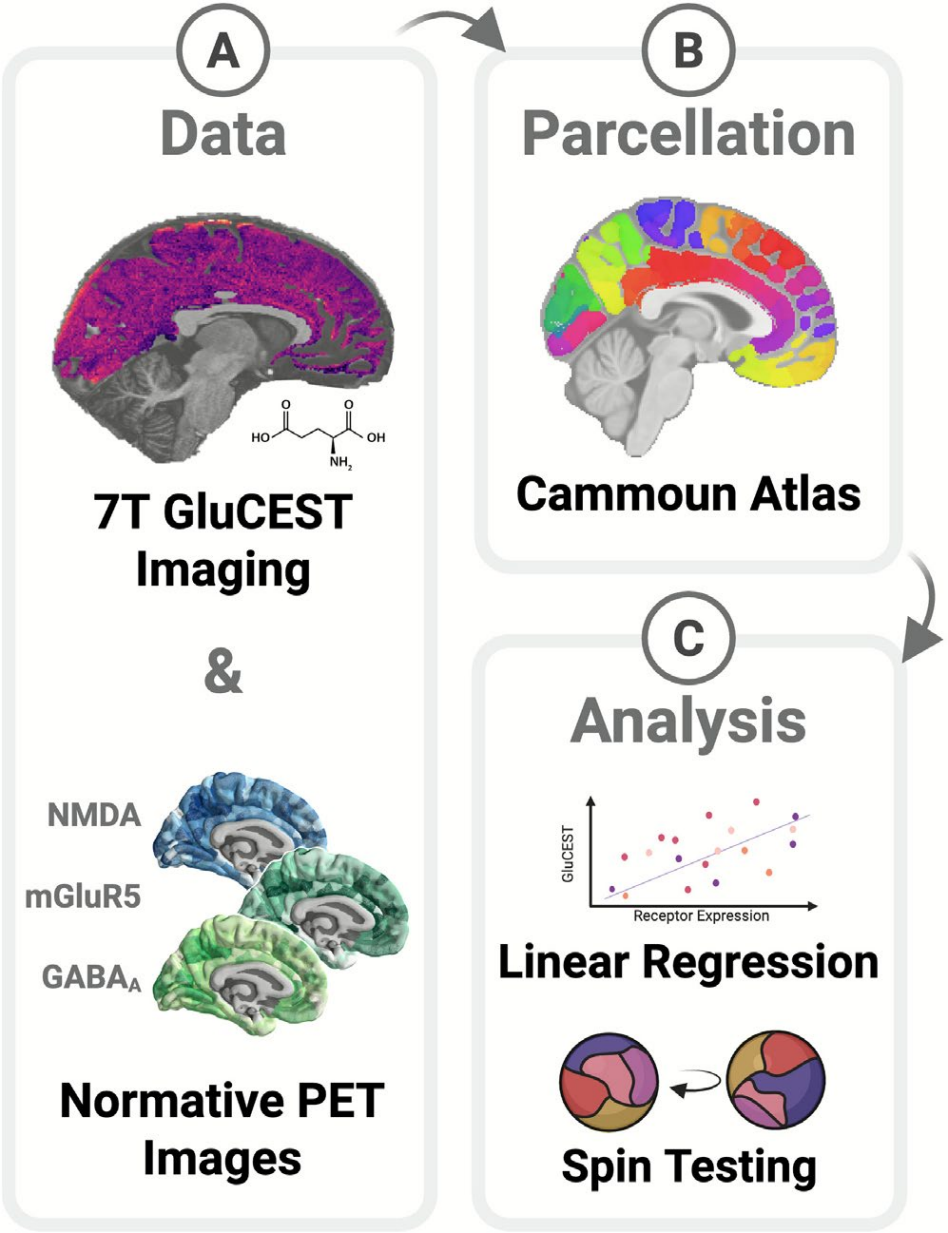
Multimodal imaging analysis pipeline. (A) *Upper panel*: Processed 7T GluCEST image (parasagittal slice, right hemisphere; 5 mm thickness, 1 × 1 mm in‐plane resolution). GluCEST data were acquired in 86 individuals. *Lower panel*: Surface plots of the three PET receptor maps analyzed. These normative data were obtained from the publicly available Neuromaps database. (B) The GluCEST and PET datasets were parcellated with the Cammoun et al. (2012) 500 atlas. (C) Parcel‐wise spatial correlations between GluCEST and receptor density were assessed via linear regression; spin‐tests were used to generate autocorrelation‐preserving null models for empirical significance testing.

Data were acquired on a 7T Siemens Terra scanner using a single‐channel transmit and 32‐channel receive head coil (Nova Medical, Wilmington, MA, USA). Structural imaging included T_1_‐weighted uniform (UNI) images alongside inversion (INV1 and INV2) images, obtained using the Magnetization Prepared 2 Rapid Acquisition Gradient Echoes (MP2RAGE) sequence (Marques et al. 2010) (parameters: TE = 2.94 ms, TR = 4900 ms, TI = 700/2700 ms, 192 sagittal slices, resolution = 0.9 × 0.9 × 0.9 mm^3^, matrix = 256 × 256, FOV = 230). For GluCEST imaging, a two‐dimensional, parasagittal acquisition (slice number: 1, slice thickness: 5 mm) was collected in the right hemisphere, providing complete in‐plane brain coverage (matrix size: 224 × 224) with an in‐plane spatial resolution of 1 × 1 mm^2^ (Figure 1A, top panel). The acquisition protocol for GluCEST, including B_0_ and B_1_ field mapping, adhered to previously established methods (Cai et al. 2012; Pecsok et al. 2024; Roalf et al. 2017). All data passed quality assurance for gross motion or inhomogeneity artifacts. Automated measures of head motion (mean and maximum head displacement) were estimated using previously reported methods (Roalf et al. 2016; Satterthwaite et al. 2012) and are reported in Table S2.

### 2.3 7T MRI Data Processing

CEST data were processed in accordance with previous studies (Jee et al. 2022; Pecsok et al. 2024; Roalf 2022; Roalf et al. 2017; Sydnor et al. 2021). T_1_‐weighted MP2RAGE UNI images acquired at 7T were bias field corrected using the Advanced Normalization Tools (ANTs) N4 algorithm (Avants et al. 2011). These corrected images were then used for tissue segmentation and atlas registration. Tissue segmentation maps, including a cortical gray matter mask, were generated with FSL FAST (Zhang et al. 2001). To parcellate our GluCEST data with a volumetric, anatomically derived atlas, UNI images were registered to the MNI152 nonlinear T_1_ template of the Cammoun500 2012 atlas (Cammoun et al. 2012), an extension of the Lausanne 2008 parcellation scheme, using ANTs' symmetric diffeomorphic normalization approach (Avants et al. 2011).

GluCEST images (Figure 1A, upper panel) quantify GluCEST contrast (%) in each voxel to represent local Glu levels (Cai et al. 2012). Image processing for GluCEST involved B_0_ and B_1_ field inhomogeneity corrections using in‐house python‐based software (pyGluCEST) (Jee et al. 2022; Roalf 2022). Voxels with B_0_ offsets exceeding ±1 ppm or relative B_1_ values outside the range of 0.3–1.3 were excluded from analysis (Roalf et al. 2017). Voxels identified as cerebrospinal fluid or volumes that had fewer than 20 voxels were excluded from further analysis. After parcellation, parcel‐wise GluCEST data for each subject were normalized (z‐score).

### 2.4 Neuromaps Data & Processing

The Neuromaps database includes the neurotransmitter density maps from PET images of over 1200 healthy individuals and includes 19 different neurotransmitter receptors and transporters for nine different neurotransmitter systems (Hansen et al. 2022; Markello et al. 2022). After downloading the relevant maps for our study (NMDA (Galovic et al. 2021), mGluR5 (DuBois et al. 2016; Hansen et al. 2022; Smart et al. 2019), GABA_A_ (Dukart et al. 2018; Nørgaard et al. 2021), and 5HT2_A_ (Beliveau et al. 2017; Savli et al. 2012) as a control; see Supplement for information on radioactive ligands), PET maps were resampled to match the resolution of the GluCEST data. Then, following the protocol outlined by Hansen et al. (2022), PET data were parcellated using the Cammoun500 2012 atlas. Parcellated values for each map were then normalized (z‐score) across parcels. Finally, individual PET maps were combined using a weighted average to create composite receptor maps for analysis (Figure 1A, lower panel). This approach standardized data across studies while preserving regional signal patterns.

### 2.5 Allen Human Brain Atlas Data and Processing

Provided by the Allen Human Brain Atlas (AHBA, https://human.brain‐map.org; (Hawrylycz et al. 2012)). Data were processed with the abagen toolbox (version 0.1.4+15.gdc4a007; https://github.com/rmarkello/abagen; (Markello et al. 2021)) using a 1015‐region volumetric atlas in MNI space. To increase spatial coverage, tissue samples were mirrored bilaterally across the left and right hemispheres (Romero‐Garcia et al. 2018). Samples were assigned to brain regions in the provided atlas if their MNI coordinates were within 2 mm of a given parcel. If a brain region was not assigned a tissue sample based on the above procedure, every voxel in the region was mapped to the nearest tissue sample from the donor in order to generate a dense, interpolated expression map. The average of these expression values was taken across all voxels in the region, weighted by the distance between each voxel and the sample mapped to it in order to obtain an estimate of the parcellated expression values for the missing region. All tissue samples not assigned to a brain region in the provided atlas were discarded. See Supplement for additional details regarding AHBA data acquisition and processing.

### 2.6 Integration of GluCEST, PET, and AHBA Data

After GluCEST and PET data were normalized and parcellated using the Cammoun500 2012 atlas, we assigned each parcel's 2D GluCEST value uniformly across its entire 3D volume to enable direct comparison with 3D PET data and AHBA data. Parcels were included in main analyses if > 60% of participants contributed GluCEST data to that parcel. Sensitivity analyses were performed with alternative thresholds. Parcels included in the primary analysis are listed in Table S3, while the number of subjects with data for each parcel is reported in Table S4. Parcels were also assigned to cytoarchitecturally defined von Economo regions for post hoc regional analyses with the PET data (Figure 1B, lower panel).

### 2.7 Statistical Analysis

Student's t‐tests and chi‐square tests were used for group demographic comparisons where appropriate. Parcel‐wise associations between GluCEST levels and PET receptor density were assessed across all participants using multiple linear regression (see Figure 1C, upper panel). Statistical significance was evaluated using the Vázquez‐Rodriguez spin test, a permutation method that accounts for spatial autocorrelation by rotating one cortical map relative to the other to generate a null distribution of correlations (Vázquez‐Rodríguez et al. 2019).

Sensitivity analyses were conducted to address potential confounding by age, sex, diagnostic status, motion, and missingness. First, GluCEST values were corrected for age, sex, and diagnosis prior to regression and spin testing. Next, dimensional clinical scales were included in place of categorical diagnoses. Lastly, analyses were run after correcting for motion parameters (mean and maximum displacement during GluCEST acquisition). The main analyses were also repeated in the replication GluCEST dataset (Table S1) and at varying parcel inclusion thresholds (20%, 40%, 60%, 80%) to account for potential bias from selective missingness. Lastly, GluCEST‐receptor trends were reevaluated using average parcel‐level GluCEST values across all participants to reduce potential bias from individual‐level variability. For this analysis, Pearson correlations were computed, and statistical significance was again evaluated using the Vázquez‐Rodriguez spin test. Resulting *p*‐values were subsequently adjusted for multiple comparisons across the four receptor correlations using the Benjamini–Hochberg false discovery rate (FDR) procedure, with significance set at q < 0.05.

Exploratory interaction analyses were conducted to evaluate associations between GluCEST and glutamatergic receptors across cytoarchitecturally defined von Economo regions (VE regions; Figure S1). Group‐averaged GluCEST data were used for these analyses, as spin tests were not well‐suited for regional comparisons. Sensitivity analyses were performed at varying parcel inclusion thresholds (20%, 40%, 60%, 80%) and in the replication dataset. To further explore regional GluCEST‐receptor trends, post hoc analyses were conducted, again in group‐level data. Separate Pearson correlations were computed for each receptor within each von Economo region. Again, sensitivity analyses were implemented. All analyses were conducted in Python and R.

Lastly, as an exploratory analysis, we used AHBA data to investigate the spatial correlation between GluCEST and the expression of genes associated with NMDA (GRIN1, GRIN2A), mGluR5 (GRM5), and GABA_A_ (GABRA1, GABRG2), as well as the genes encoding glutaminase (GLS) and glutamine synthetase (GLUL), which are primary enzymes for the synthesis and metabolism of Glu. As with the receptor data, parcel‐wise associations between GluCEST levels and gene expression were assessed across all participants using multiple linear regression (see Figure 1C, upper panel). Statistical significance was evaluated using the Vázquez‐Rodriguez spin test (Vázquez‐Rodríguez et al. 2019). Again, thorough sensitivity testing was performed to address potential confounding by age, sex, diagnostic status, motion, and missingness, and FDR correction was employed to correct for multiple comparisons.

## 3 Results

### 3.1 Participant Characteristics

The clinical and demographic information is presented in Table 1.

**TABLE 1:** Demographic and clinical characteristics of participants across clinical subgroups.

	HC	PRO/CHR	PSY	*p*‐value
	(*N* = 34)	(*N* = 31)	(*N* = 21)	
Age				
Mean (SD)	22.0 (2.89)	21.5 (3.52)	25.9 (3.32)	< 0.001
Sex				
F	15 (44.1%)	14 (45.2%)	10 (47.6%)	0.99
M	19 (55.9%)	17 (54.8%)	11 (52.4%)	
Race				
Asian	4 (11.8%)	2 (6.5%)	2 (9.5%)	0.81
Black or African American	10 (29.4%)	15 (48.4%)	9 (42.9%)	
More than one race	2 (5.9%)	4 (12.9%)	3 (14.3%)	
White	18 (52.9%)	10 (32.3%)	7 (33.3%)	
Positive symptoms^a^				
Mean (SD)	1.68 (1.82)	7.40 (3.81)	18.1 (6.43)	< 0.001
Negative symptoms^a^				
Mean (SD)	1.32 (1.36)	7.50 (5.26)	11.3 (3.72)	< 0.001
Disorganized symptoms^a^				
Mean (SD)	0.44 (0.99)	3.37 (1.96)	6.00 (3.22)	< 0.001

*Note:* Participants were categorized as healthy controls (HC), clinical high‐risk or prodromal psychosis (PRO/CHR), or psychosis spectrum disorder (PSY). Groups did not differ significantly in sex or racial composition (*p* > 0.80) but differed significantly in age and symptom severity (*p* < 0.001 for all symptom domains). Symptom scores reflect SIPS ratings within each domain. Values are reported as mean (standard deviation) for continuous variables and count (percentage) for categorical variables.

^a^ Subscales from the Structured Interview for Psychosis‐Risk Syndromes (SIPS). SIPS data were missing for one participant in the PRO/CHR and three in the PSY group.

### 3.2 GluCEST‐Receptor Associations

**FIGURE 2:**
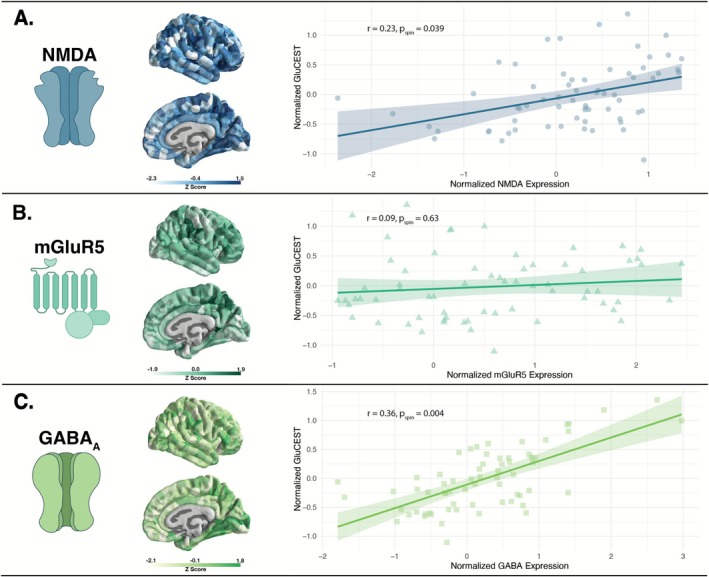
Association between GluCEST and receptor density. Each plot depicts the association between normalized GluCEST contrast and normalized receptor density as measured by normative PET maps. (A) NMDA expression was positively associated with GluCEST (*r* = 0.23, p_spin_ = 0.04). (B) There was no significant association between mGluR5 and GluCEST (*r* = 0.09, p_spin_ > 0.05). (C) There was a strong positive association between GABA_A_ and GluCEST (*r* = 0.36, p_spin_ = 0.004).

There was a strong positive association between GluCEST and both NMDA (*r* = 0.23, p_spin_ = 0.039) and GABA_A_ (*r* = 0.36, p_spin_ = 0.004) (Figure 2A,C), but not for mGluR5 (*r* = 0.09, p_spin_ = 0.63) (Figure 2B). No significant correlation was found in the control receptor, 5HT2_A_ (Figure S2). Similar trends were also identified in our reliability replication dataset (Figure S3) and at various parcel inclusion thresholds (see Data S1). The linear model showed no significant effect for age, sex, or diagnosis. Separate models incorporating SIPS subscales (positive, negative, and disorganized) as well as motion parameters (mean or maximum displacement) yielded consistent results. An additional sensitivity analysis that investigated the GluCEST‐receptor trends using group averaged parcel‐level data also yielded consistent results (see Data S1).

### 3.3 GluCEST‐Receptor Associations Across von Economo Regions

Next, we examined the relationship between GluCEST and receptor densities (NMDA, mGluR5, GABA_A_) across von Economo regions using an exploratory linear interaction model (see Figure S4) with group‐level data. There was an interaction between von Economo region and mGluR5 (F(1,57) = 12.7) and GABA_A_ (F(1,57) = 2.7), indicating differences across von Economo regions. There was no substantial interaction for NMDA (F(1,57) = 0.3). A similar pattern of results was found in the reliability replication dataset (Figure S5) and when correcting for age, sex, and diagnostic status.

**FIGURE 3:**
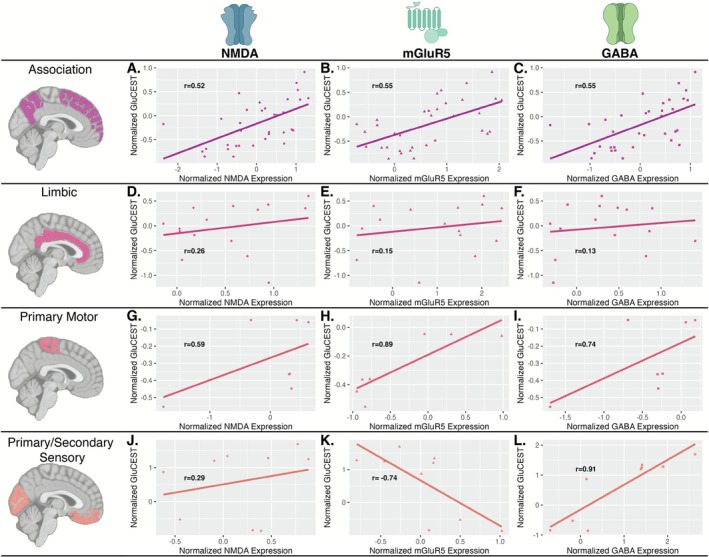
Association between GluCEST and receptor density across Von Economo‐defined cortical regions. Each plot shows the relationship between normalized GluCEST contrast and receptor density (as measured by normative PET maps) within different cortical zones. For NMDA, mGluR5, and GABA_A_, a positive GluCEST‐receptor association was found in the association cortex (A–C), limbic cortex (D–F), and primary motor cortex (G–I). In the primary/secondary sensory cortex (J–L), GluCEST showed a positive association with GABA_A_ and NMDA and a negative association with mGluR5.

Post hoc analysis was performed to further explore GluCEST‐receptor trends across von Economo regions by calculating Pearson r values for each association within each region (Figure 3). Positive GluCEST‐receptor trends were identified in the association, primary motor, and primary/secondary sensory cortices, while a negative GluCEST‐mGluR5 correlation was identified exclusively in the primary/secondary sensory cortex. These findings were replicated in the reliability dataset (Figure S6), and remained consistent after correcting for age, sex, and diagnostic status. Most trends also held across varying parcel inclusion thresholds.

### 3.4 GluCEST‐Gene Expression Associations

**FIGURE 4:**
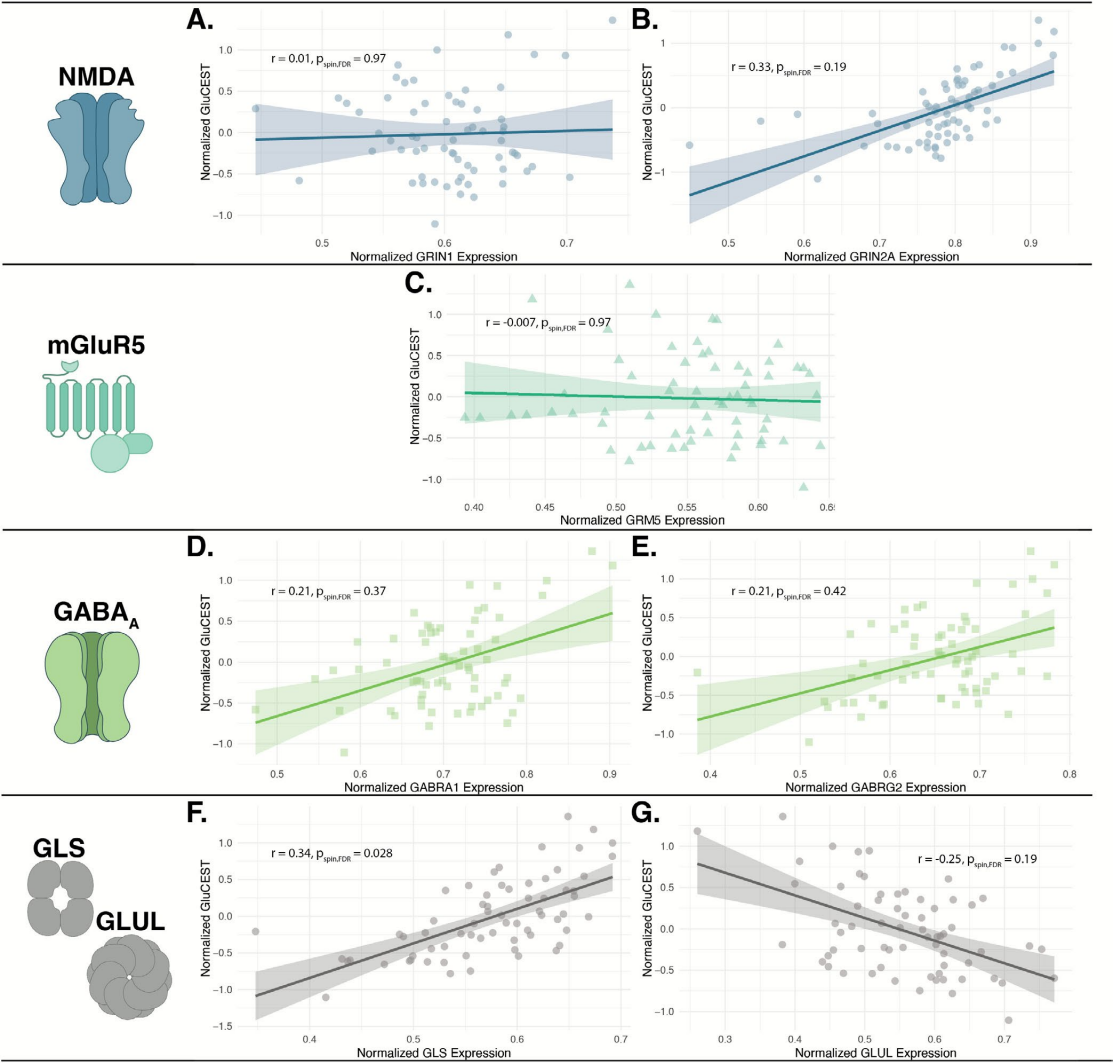
Association between GluCEST and AHBA derived gene expression. Each plot depicts the association between normalized GluCEST contrast and normalized gene expression as measured by the Allen Human Brain Atlas (AHBA). There was a significant association between GluCEST and the expression of GLS (*r* = 0.34, p_spin_ = 0.028). All other associations were not statistically significant.

There was a positive association between GluCEST and expression of GLS (Figure 4F; *r* = 0.34, p_spin_ = 0.028). Although not statistically significant, GluCEST showed positive associations with GRIN2A, GABRA1, and GABRG2 and a negative association with GLUL. Trend‐level associations with GRIN2A and GLUL were observed prior to FDR correction (see Data S1). Similar results were found in our reliability replication dataset (Figure S6), with group‐level data and at various parcel inclusion thresholds (see Data S1). The linear model showed no significant effect for age, sex, or diagnosis. Separate models incorporating SIPS subscales (positive, negative, and disorganized) as well as motion parameters (mean or maximum displacement) yielded consistent results.

## 4 Discussion

The present study demonstrates a robust positive spatial convergence between GluCEST levels and the density of NMDA and GABA_A_ receptors, but not mGluR5 receptors. This association indicates that in vivo brain Glu concentrations are, on average, higher in regions where these ionotropic glutamatergic and GABAergic receptors are more densely expressed, as measured by PET imaging. Importantly, associations were not affected by diagnostic status or psychosis spectrum symptom severity scores, indicating that GluCEST‐receptor trends in our transdiagnostic cohort were robust regardless of symptom severity. Exploratory analyses showed that GluCEST‐receptor associations varied across von Economo regions. Thus, it is possible that local cytoarchitectural milieu influences the spatial coupling between Glu levels and receptor density. In parallel, our exploratory analysis of transcriptomic data revealed gene‐level associations between GluCEST and select glutamatergic pathway genes. These findings suggest that molecular expression patterns, albeit measured at a different level of organization, broadly converge with the receptor density associations identified here. To our knowledge, this is the first study to characterize the spatial correlation between in vivo GluCEST levels and receptor density, providing novel insights into the spatial organization of glutamatergic signaling in the human brain that will allow future GluCEST studies to yield deeper knowledge of glutamatergic function and dysfunction in basic and translational contexts. In turn, the convergence with PET‐based Neuromaps highlights their potential to approximate regionally specific glutamate distribution.

Extant literature provides a plausible neurobiological framework for understanding the overall associations we identified between GluCEST levels and the expression of NMDA, mGluR5, and GABA_A_. The spatial association of GluCEST with NMDA receptor density is expected given that NMDA is a primary ionotropic receptor for Glu that is vital for glutamatergic neurotransmission (Chou et al. 2024; Pagano et al. 2021). Higher synaptic Glu concentrations have been shown to enhance not only NMDA receptor activity (Chalifoux and Carter 2011) but also NMDA receptor density: the binding of Glu to NMDA receptors is critical for the receptor's surface trafficking (She et al. 2012). The molecular coupling between Glu and NMDA receptor density and functioning likely underlies the macroscopic spatial correlation observed in the present study.

The strong positive association between the spatial overlap of GluCEST signal and GABA_A_ expression underscores the tight coupling between excitatory and inhibitory systems in the brain, which aligns with previous literature. While GABA_A_ receptors can inhibit Glu release (Long et al. 2009), other studies have reported that high Glu levels can stimulate GABA_A_ expression through direct binding of Glu to GABA_A_ subunits (Stelzer and Wong 1989; Wen et al. 2022). Higher GABA_A_ expression in glutamate‐rich regions may serve a neuroprotective role, preventing excitotoxicity (Bayón‐Cordero et al. 2022; Wen et al. 2022). Alternatively, tight coupling between GluCEST and GABA_A_ expression may reflect extensive metabolic crosstalk between these neurotransmitter systems (Sears and Hewett 2021; Wen et al. 2022). Because GluCEST does not distinguish between synaptic and extra‐synaptic glutamate, regions rich in synaptic GABA_A_ may exhibit high GluCEST due to higher Glu concentrations in other cellular compartments. While the precise mechanisms remain to be elucidated, the spatial coupling between GluCEST and GABA_A_ receptor density likely reflects a fundamental homeostatic balance between excitatory and inhibitory signaling in the brain.

The null association between GluCEST and mGluR5 also fits the broader literature. First, several prior ^1^H‐MRS‐PET studies reported no significant correlations between local PET‐derived mGluR5 density and Glu or Glx levels within the same region (Bdair et al. 2024; De Laat et al. 2018; Martinez et al. 2014). As a metabotropic receptor, mGluR5 mediates excitatory Glu signaling more indirectly by regulating surface trafficking of NMDA receptor subunits (Jin et al. 2015) and potentiating AMPA‐mediated neurotransmission (Loerwald et al. 2014). mGluR5 activation can also induce Ca^2+^ oscillations in astrocytes, which can trigger glutamate release and subsequent NMDA receptor activation on nearby neurons (D'Ascenzo et al. 2007; Nakahara et al. 1997). These mechanisms are more indirectly yoked to Glu fluctuations, which may explain why we found no significant spatial coupling between GluCEST and mGluR5 across all parcels. Alternatively, as discussed below, GluCEST‐mGluR5 coupling may vary more drastically across cytoarchitectural regions, leading to a weaker overall association with GluCEST.

For all receptors, it is plausible that synaptic density may have contributed to the measured spatial correlation between GluCEST and receptor density, as regions with higher synaptic density would be expected to have elevated levels of most neurotransmitters and neurotransmitter receptors. Supporting this idea, recent studies show that presynaptic density—as measured by synaptic vesicle glycoprotein 2 A (SV2A) PET—is correlated with PET‐measured mGluR5 availability in Alzheimer's patients (Wang et al. 2024). Additionally, a study employing PET and ^1^H‐MRS found that SV2A levels were correlated with local Glu/Cr (glutamate relative to creatine) levels in healthy volunteers within the left hippocampus and anterior cingulate cortex, with diminished or absent correlations in patients with schizophrenia (Onwordi et al. 2021). Future studies would benefit from integrating SV2A measurements to further elucidate these relationships.

Our results revealed distinct GluCEST‐receptor trends across von Economo regions, suggesting that cytoarchitectural context may modulate the interplay between receptors and neurotransmitters. von Economo regions differ in cortical structure and signaling priorities. For example, in the association cortex, the thick layer II/III facilitates intracortical communication and long‐range connectivity, supporting higher‐order information integration (Douglas and Martin 2004; Larkum 2013). In contrast, the visual cortex, which lies within the primary/secondary sensory von Economo region, features a prominent layer IV specialized for receiving dense glutamatergic input from the lateral geniculate nucleus of the thalamus (Callaway 2004; Harris and Shepherd 2015). Post hoc analyses highlighted particularly distinct patterns in the primary/secondary sensory cortex, potentially due to region‐specific receptor roles. For example, NMDA receptors dominate excitatory signaling within the dorsolateral prefrontal region of the association cortex, whereas AMPA receptors play a more prominent role in the visual cortex (Yang et al. 2018). NMDA is also involved in experience‐dependent plasticity processes unique to the visual cortex (Fong et al. 2020; Quinlan et al. 1999; Rodriguez et al. 2019). GABA_A_ function is similarly context dependent. In the primary visual cortex, GABA_A_ receptors are found on presynaptic terminals of both glutamatergic and GABAergic neurons, while they are primarily postsynaptic in other cortical areas (Wang et al. 2019). The positive associations between GluCEST and both GABA_A_ and NMDA in the primary/secondary sensory cortex may reflect the unique signaling demands of this highly specialized region.

Notably, mGluR5 expression was negatively associated with GluCEST exclusively in the primary/secondary sensory cortex. This pattern was distinct from the positive mGluR5 trends in all other regions and the GluCEST‐NMDA and ‐GABA_A_ trends in this region. This negative association may reflect mGluR5's developmental and functional role in the visual cortex. Previous studies in mice show that mGluR5 expression in the visual cortex peaks in the early postnatal period, coinciding with critical periods of synaptic and ocular dominance column plasticity (Sidorov et al. 2015). It is possible that the process leading to decreased mGluR5 expression also affects the spatial covariance between mGluR5 and Glu in this region—it would be interesting to investigate this trend in a neonatal or developmental cohort. In the mature visual system, mGluR5 activates fast‐spiking GABAergic neurons (Sarihi et al. 2008) and contributes to an input‐specific metaplasticity process unique to the visual cortex (Chokshi et al. 2019; Tsanov and Manahan‐Vaughan 2009). Perhaps the negative spatial correlation between mGluR5 and GluCEST in this region is related to the receptor's distinct role in this uniquely plastic cortex. It is possible that the negative correlation between mGluR5 and GluCEST in the visual cortex represents an adaptive uncoupling, wherein a preponderance of receptors is needed for high sensitivity responses to small amounts of Glu and a dearth of receptors moderates signal amplification in regions with high Glu levels. This interpretation aligns with evidence that mGluR5 is downregulated in high‐glutamate contexts to prevent excitotoxicity (Balázs et al. 1997; Crabbé et al. 2019; Lee and Parpura 2016). Notably, our prior work has demonstrated that GluCEST levels in the visual cortex are particularly high (Pecsok et al. 2022); the negative correlation observed in this region may reflect a protective mechanism against excitotoxicity or a means of maintaining high‐sensitivity signaling to Glu. It is also worth considering that our participants were scanned at rest with eyes either open or closed. As a result, visual input may have impacted the GluCEST signals in the primary/secondary sensory cortex and affected the spatial coupling we measured with receptor density. Prior studies have also demonstrated that the visual cortex has distinct gene and receptor density profiles compared to other brain regions (Eickhoff et al. 2008; Gomez et al. 2019). Multiple factors impacting cytoarchitecture, metabolism, and neurotransmission likely contributed to the distinct GluCEST‐receptor trends we identified in this region. Further research is needed to better characterize region‐specific transmitter‐receptor spatial coupling.

In parallel with the receptor density analyses, we conducted an exploratory analysis to examine the covariation between GluCEST and transcriptomic data derived from the AHBA, providing an additional layer of biological validation. We observed a positive association with GRIN2A, which encodes an NMDA receptor subunit, though this trend was not statistically significant. No association was detected with GRIN1, the obligatory subunit of the NMDA receptor. This partial overlap somewhat mirrors the receptor‐level findings for NMDA, where a positive spatial association was found. The divergence between receptor and gene expression findings may reflect differential regional expression patterns across NMDA receptor subunits, where GRIN2A variation more closely captures spatial heterogeneity in NMDA expression and Glu levels. Consistent with the mGluR5 PET data, no significant correlation was observed between GluCEST and the expression of GRM5, which encodes for the mGluR5 protein. This may stem from the modulatory role of mGluR5, which is less directly coupled to basal Glu concentrations and flux than ionotropic receptors. While associations between GluCEST and both GABRA1 and GABRG2, which encode GABA_A_ receptor subunits, were not statistically significant, both showed positive trends with GluCEST that were directionally consistent with the receptor density findings. These weaker correlations may reflect limitations of transcriptomic atlases, such as sparse donor sampling and imperfect mapping to protein expression, especially in the context of field of view limitations conferred by 2D GluCEST.

In addition to receptor‐related genes, we examined GLS, which encodes glutaminase, and GLUL, which encodes glutamine synthetase. GluCEST was positively associated with GLS expression, suggesting that regions with higher glutaminase transcription—and thus greater capacity for Glu synthesis—exhibit elevated GluCEST signal. In neurons, glutaminase expression is upregulated during heightened synaptic demand, which may partly account for this correlation (Hayashi 2018; Schousboe et al. 2013). While the association between GLUL and GluCEST was not statistically significant, the negative trend suggests that regions with higher GluCEST signal also show capacity for converting glutamate to glutamine. Together, these exploratory findings suggest that gene‐level expression patterns converge with receptor‐level results, linking GluCEST signal not only to receptor distribution but also to molecular pathways of glutamate metabolism and homeostasis.

Across all analyses, we found no impact of diagnostic status or psychosis spectrum symptom severity score on GluCEST‐receptor or GluCEST‐gene expression trends, and follow‐up reliability replication analyses revealed similar associations across clinical groups. This finding suggests that the robust spatial correlations we identified overshadow potential differences related to psychopathology, at least as defined by diagnostic labels and symptom severity scores in this study. This absence of diagnosis effects contrasts with a previous study that employed simultaneous PET‐MRS to identify a negative association between hippocampal NMDA receptor density and striatal glutamate levels in patients with first‐episode psychosis—an effect absent in healthy controls (Beck et al. 2022). However, their study utilized ^1^H‐MRS rather than GluCEST and did not assess direct spatial correlations. Despite well‐documented Glu dysregulation in psychosis (Kruse and Bustillo 2022; Merritt et al. 2023), homeostatic mechanisms may preserve GluCEST‐receptor coupling at the macroscopic level.

While this study provides novel insights into the spatial correlations between GluCEST‐measured Glu levels and PET‐derived receptor density, several limitations should be acknowledged. Although GluCEST offers unprecedented sensitivity and spatial resolution and high reliability (Cember et al. 2022; Nanga et al. 2018), it is nonetheless limited by the acquisition of a single parasagittal slice—restricting whole‐brain coverage and yielding data from only part of each parcel that may not be fully representative. Given the limited spatial resolution of Neuromaps, we chose a conservative parcellated approach for our analyses to limit potential bias. Future studies could leverage recent advances in 3D GluCEST to enable full‐brain coverage (Jacobs et al. 2022). Another limitation of GluCEST is that about 30% of signal may be attributed to contamination from the creatine, GABA, and other macromolecules (Cai et al. 2012). While this could have contributed to the observed association between GluCEST and GABA_A_ receptor density, the moderate effect sizes we measured are unlikely to be fully explained by this confound. Future investigations could leverage high‐resolution magnetic resonance spectroscopy imaging (MRSI) alongside GluCEST to systematically quantify transmitter–receptor relationships across a broader spectrum of metabolites. In addition, preclinical studies have demonstrated that GABACEST can noninvasively map GABA distribution in animal models (Shen et al. 2025; Yan et al. 2016). Upon successful adaptation to human imaging, GABACEST could complement GluCEST by providing a fuller picture of excitation–inhibition dynamics and transmitter–receptor spatial associations. Another necessary limitation of our study was the reliance on normative receptor and genetics data rather than subject‐specific maps or data acquired in participants across the psychosis spectrum. Given documented glutamatergic receptor dysfunction in psychosis populations (Nakazawa and Sapkota 2020), PET‐derived receptor maps from clinical groups may exhibit divergent spatial correlations with GluCEST. Future investigations with larger sample sizes and within‐subjects PET data will be essential to explore this issue further.

The scope of this study was intentionally narrow, focusing on a select few neurotransmitter receptors—NMDA, mGluR5, and GABA_A_—due to their relevance to our hypothesis‐driven approach and their availability in Neuromaps. While a PET‐derived AMPA brain annotation is not yet available, its inclusion in future studies will be crucial given its central role in glutamatergic signaling. Glu interacts with many neurotransmitter receptors, and future studies could expand upon the present work to examine spatial covariance between GluCEST and other receptor maps for a more comprehensive understanding of how Glu is integrated into diverse neurotransmitter systems. To extend beyond receptor‐level analyses, we incorporated Allen Human Brain Atlas (AHBA) gene expression data as an additional layer of biological validation. Again, a focused approach was adopted, and we examined a limited set of receptor‐related genes along with the primary enzymes for Glu synthesis and catabolism. Future work could broaden the gene set or apply enrichment analyses and other transcriptomic approaches to yield additional insights. Importantly, the AHBA dataset itself has limitations. Because of its relative sparsity of coverage and small sample size, gene expression results should be interpreted as convergent support for the PET‐based analyses rather than as a standalone validation of GluCEST. Moving forward, integrating transcriptomic, receptor density, and GluCEST data collected within the same individuals will be essential for establishing a more mechanistic, multilevel framework for glutamatergic imaging. Incorporating synaptic density and other structural and functional maps could provide further insights into the likely complex relationship between GluCEST and neurotransmitter receptor density. Lastly, complementary glutamate imaging methods such as ^1^H‐MRS and MRSI offer valuable opportunities to corroborate the present findings and assess spatial correlations with other neurotransmitters.

The present findings may be of translational value. While prior studies have established that GluCEST correlates with ^1^H‐MRS data (Bagga et al. 2018; Sydnor et al. 2021), this study is the first to leverage standardized receptor maps and in vivo GluCEST imaging to demonstrate these GluCEST‐receptor associations in the human brain. These spatial correlations provide a novel framework for interpreting GluCEST findings, allowing for more nuanced insights into glutamatergic function and dysfunction across translational contexts. Moreover, this study demonstrates the utility of Neuromaps as a complementary tool for neuroimaging studies that can enhance the interpretation of diverse imaging modalities. Future studies could expand upon this work by exploring additional maps and imaging modalities that may spatially correlate with GluCEST. Lastly, implementing PET and GluCEST within subjects across diverse healthy and clinical populations will be crucial for further characterizing how GluCEST‐receptor associations vary across disease states and cytoarchitectural brain regions. By combining two powerful neuroimaging approaches, this study provides a new avenue for investigating glutamatergic signaling in the human brain and lays a foundation for future discoveries in both basic and clinical neuroscience.

## Funding

This work was supported by the University of Pennsylvania, P30 AG072979; National Institutes of Health, R01AG063869, RF1AG087306; National Institute of Biomedical Imaging and Bioengineering, P41EB029460; National Institute of Mental Health, F30MH136690, R01MH112847, R01MH119185, R01MH120174, R01MH123550, R01NS112274, R56AG066656.

## Supporting information


**Table S1:** Demographics of replication dataset: Demographics are presented for the reliability replication dataset. These participants are a subset of the main cohort who had a second GluCEST acquisition.
**Table S2:** Motion data in main dataset. Scanning quality during the 7T GluCEST acquisition was high in both groups. Mean head displacement was higher in the PSY group than the HC group (*p* < 0.05). There was no significant difference in maximum head displacement between groups.
**Table S3:** Parcels included in primary analyses. This table lists all parcels from the Cammoun500 2012 atlas included in our analyses, grouped by von Economo region. All parcels were in the right hemisphere, where the parasagittal GluCEST slab was located.
**Table S4:** Summary Information for Parcels. This table summarizes the number of parcels in each von Economo region as well as average number of participants represented in each parcel at various parcel inclusion thresholds. The inclusion threshold of 60% was used for main analyses. In all thresholds, association cortex had the greatest number of parcels.
**Figure S1:** Regional Analysis Approach. (A) Cytoarchitecturally defined von Economo regions were used for post hoc regional analyses. (B) Post hoc regional analyses were performed within von Economo regions.
**Figure S2:** GluCEST‐receptor trend in control receptor. There was no significant association between GluCEST and the density of 5HT2A (r = 0.07, pspin, FDR = 0.63).
**Figure S3:** GluCEST‐receptor trends in replication dataset. In the replication dataset (*n* = 53), there was a significant positive association between GluCEST and NMDA (r = 0.25, pspin = 0.045), mGluR5 (r = 0.10, pspin > 0.05), and GABAA (r = 0.37, pspin = 0.0006). These results are consistent with our primary analysis.
**Figure S4:** GluCEST‐Receptor trends across von Economo regions. There was no significant interaction between NMDA and von Economo region (F(3,57) = 0.28, p_FDR_ > 0.05). However, there was an interaction between von Economo region and both mGluR5 (F(3,57) = 8.1, p_FDR_ < 0.001) and GABA_A_ (F(3,57) = 2.7, p_FDR_ = 0.08) in their respective models.
**Figure S5:** GluCEST‐Receptor trends across von Economo regions in the replication dataset. There was no significant interaction between NMDA and von Economo region (F(3,57) = 0.6, p_FDR_ > 0.05). However, there was an interaction between von Economo region and both mGluR5 (F(3,57) = 12.7, p_FDR_ < 0.001) and GABA_A_ (F(3,57) = 5.6, p_FDR_ = 0.003) in their respective models.
**Figure S6:** GluCEST‐receptor trends in each von Economo region for reliability dataset. Results are shown for the replication dataset. Each plot shows the relationship between normalized GluCEST contrast and receptor density (as measured by normative PET maps) within different cortical zones. For NMDA, mGluR5, and GABAA, a positive GluCEST‐receptor association was found in the association cortex (A–C), limbic cortex (D–F), and primary motor cortex (G–I). In the primary/secondary sensory cortex (J–L), GluCEST showed a positive association with GABAA and NMDA and a negative association with mGluR5.
**Figure S7:** GluCEST‐gene trends in replication dataset. In the replication dataset (*n* = 53), there was a positive association between GluCEST and GLS (r = 0.30, pspin = 0.049). There were no significant associations with GRIN1 (r = 0.02, pspin = 0.99), GRIN2A (r = 0.27, pspin = 0.23), GRM5 (r = −0.002, pspin = 0.99), GABRA1 (r = 0.19, pspin = 0.33), GABRG2 (r = 0.18, pspin = 0.45), or GLUL (r = −0.24, pspin = 0.21). These results are consistent with our primary exploratory analysis.

## Data Availability

Research data are not shared.
