# Supplementary material for: Characterizing Spatial Associations Between GluCEST MRI and Neurotransmitter Receptor Density in the Human Cortex
Source: Hum Brain Mapp. 2025 Dec 23;46(18):e70442. doi: 10.1002/hbm.70442 (PMC12728121; doi:10.1002/hbm.70442)
Supplement: Supplementary file 1 — Table S1: Demographics of replication dataset: Demographics are presented for the reliability replication dataset. These participants are a subset of the main cohort who had a second GluCEST acquisition. Table S2: Motion data in main dataset. Scanning quality during the 7T GluCEST acquisition was high in both groups. Mean head displacement was higher in the PSY group than the HC group (p < 0.05). There was no significant difference in maximum head displacement between groups. Table S3: Parcels included in primary analyses. This table lists all parcels from the Cammoun500 2012 atlas included in our analyses, grouped by von Economo region. All parcels were in the right hemisphere, where the parasagittal GluCEST slab was located. Table S4: Summary Information for Parcels. This table summarizes the number of parcels in each von Economo region as well as average number of participants represented in each parcel at various parcel inclusion thresholds. The inclusion threshold of 60% was used for main analyses. In all thresholds, association cortex had the greatest number of parcels. Figure S1: Regional Analysis Approach. (A) Cytoarchitecturally defined von Economo regions were used for post hoc regional analyses. (B) Post hoc regional analyses were performed within von Economo regions. Figure S2: GluCEST‐receptor trend in control receptor. There was no significant association between GluCEST and the density of 5HT2A (r = 0.07, pspin, FDR = 0.63). Figure S3: GluCEST‐receptor trends in replication dataset. In the replication dataset (n = 53), there was a significant positive association between GluCEST and NMDA (r = 0.25, pspin = 0.045), mGluR5 (r = 0.10, pspin > 0.05), and GABAA (r = 0.37, pspin = 0.0006). These results are consistent with our primary analysis. Figure S4: GluCEST‐Receptor trends across von Economo regions. There was no significant interaction between NMDA and von Economo region (F(3,57) = 0.28, pFDR > 0.05). However, there was an interaction between von Eco [file HBM-46-e70442-s001.docx]

**Supplemental Material for**

**Characterizing Spatial Associations Between GluCEST MRI and Neurotransmitter Receptor density in the Human Cortex**

Maggie K. Pecsok^1^, Golia Shafei^2^, Ally Atkins^1^, Monica E. Calkins^1^, Ruben C. Gur^1^, Ravi Prakash Reddy Nanga^3^, Ravinder Reddy^3^, Melanie A. Matyi^4^, Jacquelyn Stifelman^1^, Heather Robinson^1^, Erica B. Baller^1^, Russell T. Shinohara^5^, Kosha Ruparel^1^, Kristin A. Linn^5^, Daniel H. Wolf^1^, Theodore D. Satterthwaite^2^, Corey T. McMillan^4^ & David Roalf^1^

**Methods**

|  | **HC** | **PSY** | **P-value** |
| --- | --- | --- | --- |
|  | (N=27) | (N=26) |  |
| **Age** |  |  |  |
| **Mean (SD)** | 22.0 (2.69) | 23.1 (3.37) | 0.183 |
| **Sex** |  |  |  |
| **F** | 13 (48.1%) | 9 (34.6%) | 0.471 |
| **M** | 14 (51.9%) | 17 (65.4%) |  |
| **Race** |  |  |  |
| **Asian** | 4 (14.8%) | 2 (7.7%) | 0.815 |
| **Black or African American** | 9 (33.3%) | 10 (38.5%) |  |
| **More than one race** | 2 (7.4%) | 3 (11.5%) |  |
| **White** | 12 (44.4%) | 11 (42.3%) |  |

HC: Healthy control; PSY: Psychosis Spectrum disorder group

**Table S1: Demographics of replication dataset:** Demographics are presented for the reliability replication dataset. These participants are a subset of the main cohort who had a second GluCEST acquisition.

|  | **HC** | **PRO/CHR** | **PSY** | **Overall** | **P-value** |
| --- | --- | --- | --- | --- | --- |
|  | **(N=34)** | **(N=31)** | **(N=21)** | **(N=86)** |  |
| **Motion (mean displacement)** | |  |  |  |  |
| **Mean (SD)** | 0.121 (0.0695) | 0.185  (0.149) | 0.191 (0.174) | 0.161 (0.133) | 0.04 |
| **Missing** | 1 (2.9%) | 0 (0%) | 2 (9.5%) | 3 (3.5%) |  |
| **Motion (max displacement)** | |  |  |  |  |
| **Mean (SD)** | 0.503 (0.373) | 0.707  (0.601) | 0.746 (0.698) | 0.635 (0.553) | 0.25 |
| **Missing** | 1 (2.9%) | 0 (0%) | 2 (9.5%) | 3 (3.5%) |  |

**Table S2. Motion data in main dataset.** Scanning quality during the 7T GluCEST acquisition was high in both groups. Mean head displacement was higher in the PSY group than the HC group (p<0.05). There was no significant difference in maximum head displacement between groups.

**PET ligand information**

- The NMDA receptor data were derived a study that used [18F]GE-179 PET on 29 participants to map relative receptor density in the brain (Galovic et al., 2021). [18F]GE-179 is a well-validated ligand that binds to the phencyclidine (PCP) site within the NMDA receptor ion channel, which is accessible only when the channel is in its open state; this allows [18F]GE-179 to act as a use-dependent marker of NMDA receptor activation (McGinnity et al., 2014; Vibholm et al., 2021).
- mGluR5 data were taken from 3 separate studies that utilized the [(11)C]ABP688 ligand to quantify mGluR5 distribution in the brain (DuBois et al., 2016; Hansen et al., 2022; Smart et al., 2019). [(11)C]ABP688 is a noncompetitive and highly selective antagonist for mGluR5 that has been well-validated in vivo (Ametamey et al., 2006, 2007).
- **The GABA_A_ data was derived from two prior publications that used** [11C]**flumazenil in 6 and 16 subjects, respectively to quantify GABA_A_ receptor density** (Dukart et al., 2018; Nørgaard et al., 2021)**.** [11C]**flumazenil**has been extensively used to study the distribution and density of GABA_A_ receptors in the brain. This radiotracer binds to the benzodiazepine site of the GABA_A_ ​ receptor, allowing for the visualization and quantification of receptor availability in various neurological and psychiatric conditions (Frankle et al., 2015; Syvänen et al., 2011)
- The 5HT2_A_ data was derived from two prior studies that used the [11C]Cimbi-36 and altanserin probes, respectively, to quantify 5HT2_A_ distribution (Beliveau et al., 2017; Savli et al., 2012). Both probes are highly specific to 5HT2_A_ and have been validated *in vivo* (Ettrup et al., 2014; Riss et al., 2011).

**Gene information**

- **Genes related to NMDA:**
  - The GRIN1 gene encodes the GluN1 subunit, which is the obligatory component of all canonical NMDA receptors. Two GluN1 subunits are required in every receptor tetramer, where they form the binding site for the co-agonist glycine or D-serine (Chou et al., 2022).
  - The GRIN2A gene encodes the GluN2A subunit, a predominant regulatory subunit in the mammalian forebrain (Yashiro & Philpot, 2008). Its inclusion in the receptor complex determines critical functional properties, including channel kinetics (deactivation time), magnesium block sensitivity, and pharmacological profile (Song et al., 2024; Sun et al., 2017).
- **Genes related to mGluR5:**
  - The GRM5 gene encodes the metabotropic glutamate receptor 5 protein (Corti et al., 2003; Daggett et al., 1995). As the gene encoding for the direct molecular target of the mGluR5-PET radioligand, the
    GRM5 gene expression map is the most fundamental genetic correlate for the mGluR5-PET signal.
- **Genes related to GABA_A_:**
  - GABRA1 and GABRG2 encode two essential subunits that assemble the most common GABA-A receptor isoform in the adult central nervous system (Sigel & Steinmann, 2012; Zhu et al., 2018). Their combined expression, therefore, defines the spatial distribution of the most prevalent inhibitory receptor in the brain (Stojanovic et al., 2016). The relevance of these specific subunits is further underscored by the pharmacology of GABA_A_ PET ligands. Most widely used tracers, such as derivatives of flumazenil, target the benzodiazepine (BZD) binding site (Frankle et al., 2015). This allosteric modulatory site is located at the interface between an alpha and gamma subunit, which are encoded by GABRA1 and GABRG2, respectively.
- **Additional genes of interest:**
  - The GLS gene encodes the enzyme glutaminase. In the presynaptic neuron, the enzyme glutaminase catalyzes the hydrolysis of glutamine back into glutamate, an important step in the glutamate-glutamine cycle. This newly synthesized glutamate is then available for packaging into synaptic vesicles (Cardona et al., 2015; van Kuilenburg et al., 2019). Therefore, the expression of GLS is a direct marker of a neuron's capacity to produce glutamate for neurotransmission.
  - The GLUL gene encodes glutamine synthetase (GS), an enzyme that is predominantly expressed in astrocytes (Rose et al., 2013). GS catalyzes the ATP-dependent amidation of glutamate to form glutamine. This reaction is the primary mechanism for clearing and detoxifying glutamate taken up from the synaptic cleft and produces the glutamine that is subsequently shuttled back to neurons to serve as the precursor for new glutamate synthesis (Andersen & Schousboe, 2023; Tecson et al., 2025). The expression of GLUL is therefore a marker of the brain's capacity to support high rates of glutamatergic turnover.

| **von Economo Region** | **Parcels** |
| --- | --- |
| association | frontalpole_1R, precuneus_11R, precuneus_12R, precuneus_13R, precuneus_14R, precuneus_15R, precuneus_18R, precuneus_19R, precuneus_21R, precuneus_22R, precuneus_5R, precuneus_7R, precuneus_8R, superiorfrontal_10R, superiorfrontal_12R, superiorfrontal_13R, superiorfrontal_17R, superiorfrontal_18R, superiorfrontal_1R, superiorfrontal_20R, superiorfrontal_23R, superiorfrontal_24R, superiorfrontal_25R, superiorfrontal_29R, superiorfrontal_30R, superiorfrontal_31R, superiorfrontal_32R, superiorfrontal_36R, superiorfrontal_38R, superiorfrontal_39R, superiorfrontal_40R, superiorfrontal_7R, superiorfrontal_8R, superiorfrontal_9R |
| limbic | caudalanteriorcingulate_2R, caudalanteriorcingulate_5R, isthmuscingulate_1R, isthmuscingulate_2R, isthmuscingulate_3R, isthmuscingulate_4R, isthmuscingulate_5R, posteriorcingulate_2R, posteriorcingulate_4R, posteriorcingulate_7R, posteriorcingulate_8R, posteriorcingulate_9R, rostralanteriorcingulate_1R, rostralanteriorcingulate_2R, rostralanteriorcingulate_3R |
| primary motor | paracentral_11R, paracentral_12R, paracentral_1R, paracentral_2R, paracentral_3R, paracentral_4R, paracentral_5R |
| primary sensory* | pericalcarine_3R |
| primary/secondary sensory | cuneus_3R, cuneus_5R, cuneus_6R, lingual_11R, lingual_13R, lingual_9R, medialorbitofrontal_11R, medialorbitofrontal_1R, medialorbitofrontal_9R |

*Primary sensory cortex was excluded from regional interaction models since only one parcel was represented.

**Table S3: Parcels included in primary analyses.** This table lists all parcels from the Cammoun500 2012 atlas included in our analyses, grouped by von Economo region. All parcels were in the right hemisphere, where the parasagittal GluCEST slab was located.

|  | **Inclusion Threshold** | | | | | | | |
| --- | --- | --- | --- | --- | --- | --- | --- | --- |
|  | **80%** | | **60%*** | | **40%** | | **20%** | |
| **Von Economo Region** | **Number of Parcels** | **Subjects per Parcel**  **(Mean [SD])** | **Number of Parcels** | **Subjects per Parcel**  **(Mean [SD])** | **Number of Parcels** | **Subjects per Parcel**  **(Mean [SD])** | **Number of Parcels** | **Subjects per Parcel**  **(Mean [SD])** |
| **association** | 25 | 77.84 [3.16] | 34 | 75.97 [5.74] | 36 | 73.75 [7.98] | 40 | 69.08 [16.17] |
| **limbic** | 7 | 79.71 [4.96] | 15 | 69.93 [11.29] | 21 | 62.71 [14.71] | 23 | 59.87 [16.94] |
| **primary motor** | 5 | 76.4 [3.85] | 7 | 74 [7.79] | 9 | 66 [14.84] | 10 | 62.5 [17.84] |
| **primary sensory** | 0 | NA [NA] | 1 | 57 [NA] | 4 | 49.75 [5.44] | 4 | 49.75 [5.44] |
| **primary/secondary sensory** | 1 | 76 [NA] | 9 | 62.22 [6.96] | 14 | 57.07 [10.39] | 20 | 48.25 [16.58] |

*The 60% threshold was used for main analyses.

**Table S4: Summary Information for Parcels.** This table summarizes the number of parcels in each von Economo region as well as average number of participants represented in each parcel at various parcel inclusion thresholds. The inclusion threshold of 60% was used for main analyses. In all thresholds, association cortex had the greatest number of parcels.

**
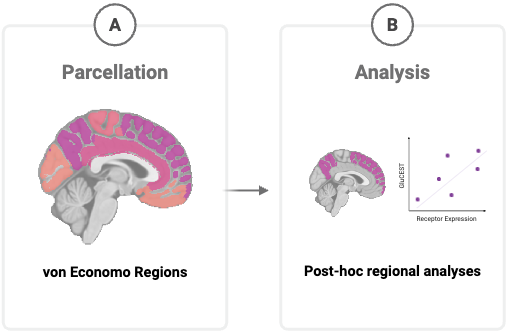
**

**Figure S1: Regional Analysis Approach A.** Cytoarchitecturally defined von Economo regions were used for post-hoc regional analyses. **B.** Post-hoc regional analyses were performed within von Economo regions.

**Allen Human Brain Atlas Detailed Methods (generated by AHBA’s abagen python package):**

First, microarray probes were reannotated using data provided by (Arnatkeviciute et al., 2019); probes not matched to a valid Entrez ID were discarded. Next, probes were filtered based on their expression intensity relative to background noise (Quackenbush, 2002), such that probes with intensity less than the background in >=50.00% of samples across donors were discarded , yielding 31,569 probes . When multiple probes indexed the expression of the same gene, we selected and used the probe with the most consistent pattern of regional variation across donors (i.e., differential stability; (Hawrylycz et al., 2015), calculated with:

$$ \Delta_{{S}}(p) = \frac{{1}}{{\binom{{N}}{{2}}}} \, \sum_{{i=1}}^{{N-1}} \sum_{{j=i+1}}^{{N}} \rho[B_{{i}}(p), B_{{j}}(p)] $$

where $ \rho $ is Spearman's rank correlation of the expression of a single probe, p, across regions in two donors $B_{{i}}$ and $B_{{j}}$, and N is the total number of donors. Here, regions correspond to the structural designations provided in the ontology from the AHBA.

The MNI coordinates of tissue samples were updated to those generated via non-linear registration using the Advanced Normalization Tools (ANTs; https://github.com/chrisfilo/alleninf). To increase spatial coverage, tissue samples were mirrored bilaterally across the left and right hemispheres (Romero-Garcia et al., 2018). Samples were assigned to brain regions in the provided atlas if their MNI coordinates were within 2 mm of a given parcel. If a brain region was not assigned a tissue sample based on the above procedure, every voxel in the region was mapped to the nearest tissue sample from the donor in order to generate a dense, interpolated expression map. The average of these expression values was taken across all voxels in the region, weighted by the distance between each voxel and the sample mapped to it, in order to obtain an estimate of the parcellated expression values for the missing region. All tissue samples not assigned to a brain region in the provided atlas were discarded.

Inter-subject variation was addressed by normalizing tissue sample expression values across genes using a robust sigmoid function (Fulcher et al., 2013):

$$ x_{{norm}} = \frac{{1}}{{1 + \exp(-\frac{{(x-\langle x \rangle)}} {{\text{{IQR}}_{{x}}}})}} $$

where $\langle x \rangle$ is the median and $\text{{IQR}}_{{x}}$ is the normalized interquartile range of the expression of a single tissue sample across genes. Normalized expression values were then rescaled to the unit interval:

$$ x_{{scaled}} = \frac{{x_{{norm}} - \min(x_{{norm}})}} {{\max(x_{{norm}}) - \min(x_{{norm}})}} $$

Gene expression values were then normalized across tissue samples using an identical procedure. Samples assigned to the same brain region were averaged separately for each donor and then across donors, yielding a regional expression matrix with 1015 rows, corresponding to brain regions, and 15,633 columns, corresponding to the retained genes.

**Results**

**
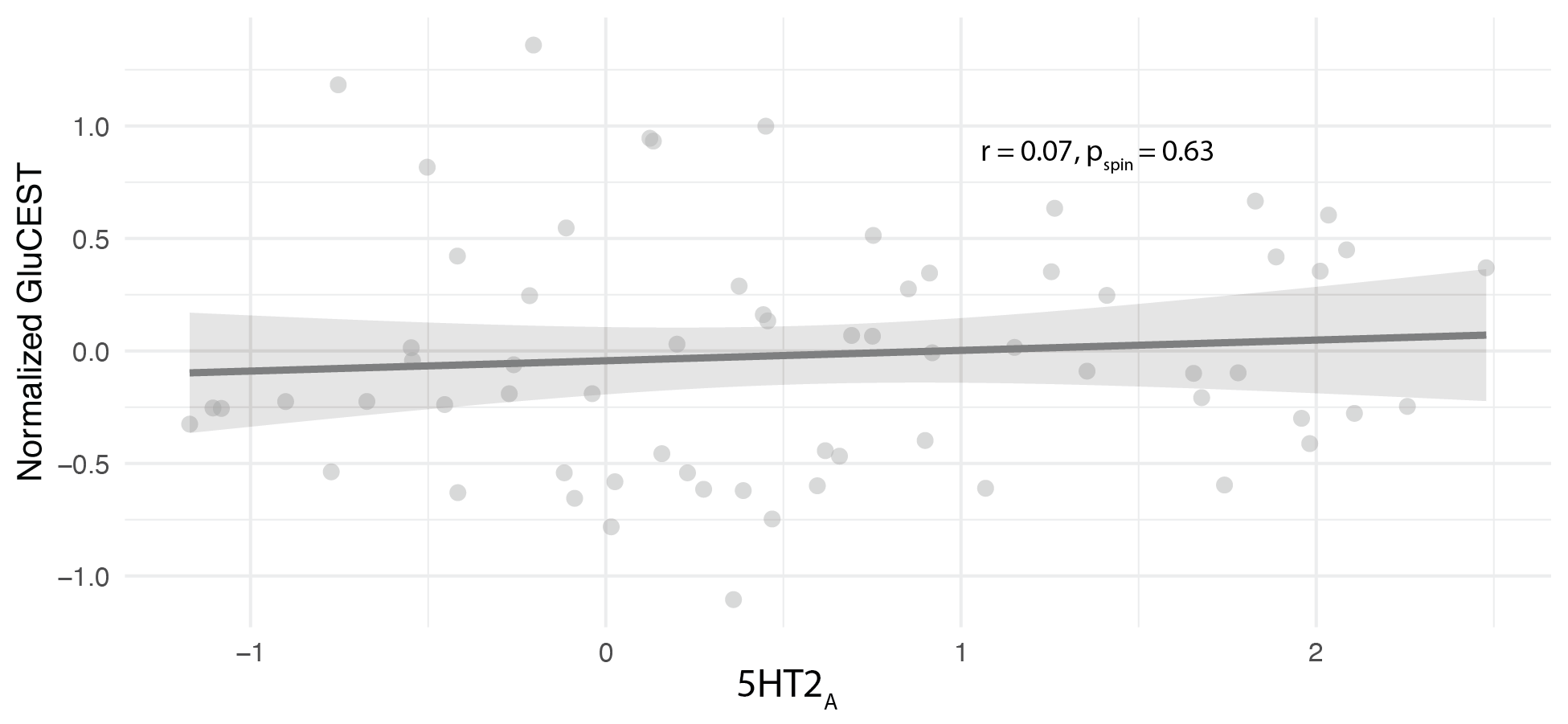
**

**Figure S2: GluCEST-receptor trend in control receptor.**

There was no significant association between GluCEST and the density of 5HT2_A_ (r = 0.07, p_spin,_ FDR = 0.63).

**Figure S3: GluCEST-receptor trends in replication dataset.**

In the replication dataset (n = 53), there was a significant positive association between GluCEST and NMDA (r = 0.25, p_spin_ = 0.045), mGluR5 (r = 0.10, p_spin_ > 0.05), and GABA_A_ (r = 0.37, p_spin_ = 0.0006). These results are consistent with our primary analysis.

**GluCEST-receptor trends corrected for potential confounding variables.**

To evaluate the robustness of parcel-wise correlations between GluCEST and receptor density, we conducted several sensitivity analyses using residualized GluCEST data. Specifically, we corrected for the following covariates in separate models:

- Age, sex, and diagnostic group
- Age, sex, and individual SIPS subscale scores (Positive, Negative, Disorganized)
- Age, sex, diagnosis, and motion parameters (mean and maximum)

Across all models, the spatial correlation results remained consistent with the primary analysis.

**GluCEST-receptor trends at various parcel inclusion thresholds:**

20% Parcel Inclusion Threshold:

- NMDA r = 0.09, p_spin_ = 0.7
- mGluR5: r = -0.04, p_spin_ = 0.7
- GABA_A_: r = 0.27, p_spin_ = 0.036
- 5HT2A: r = -0.05, p_spin_ = 0.7

40% Parcel Inclusion Threshold:

- NMDA r = 0.18, p_spin_ = 0.20
- mGluR5: r = -0.004, p_spin_ = 0.69
- GABA_A_: r = 0.33, p_spin_ = 0.002
- 5HT2A: r = -0.02, p_spin_ = 0.89

80% Parcel Inclusion Threshold:

- NMDA r = 0.27, p_spin_ = 0.006
- mGluR5: r = 0.21, p_spin_ = 0.09
- GABA_A_: r = 0.24, p_spin_ = 0.008
- 5HT2A: r = 0.17, p_spin_ = 0.21

**GluCEST-receptor density trends with means-level data**

- NMDA r = 0.40, p_spin_ < 0.0002
- mGluR5: r = 0.12, p_spin_ = 0.53
- GABA_A_: r = 0.67, p_spin_ < 0.0004
- 5HT2A: r = 0.08, p_spin_ = 0.75


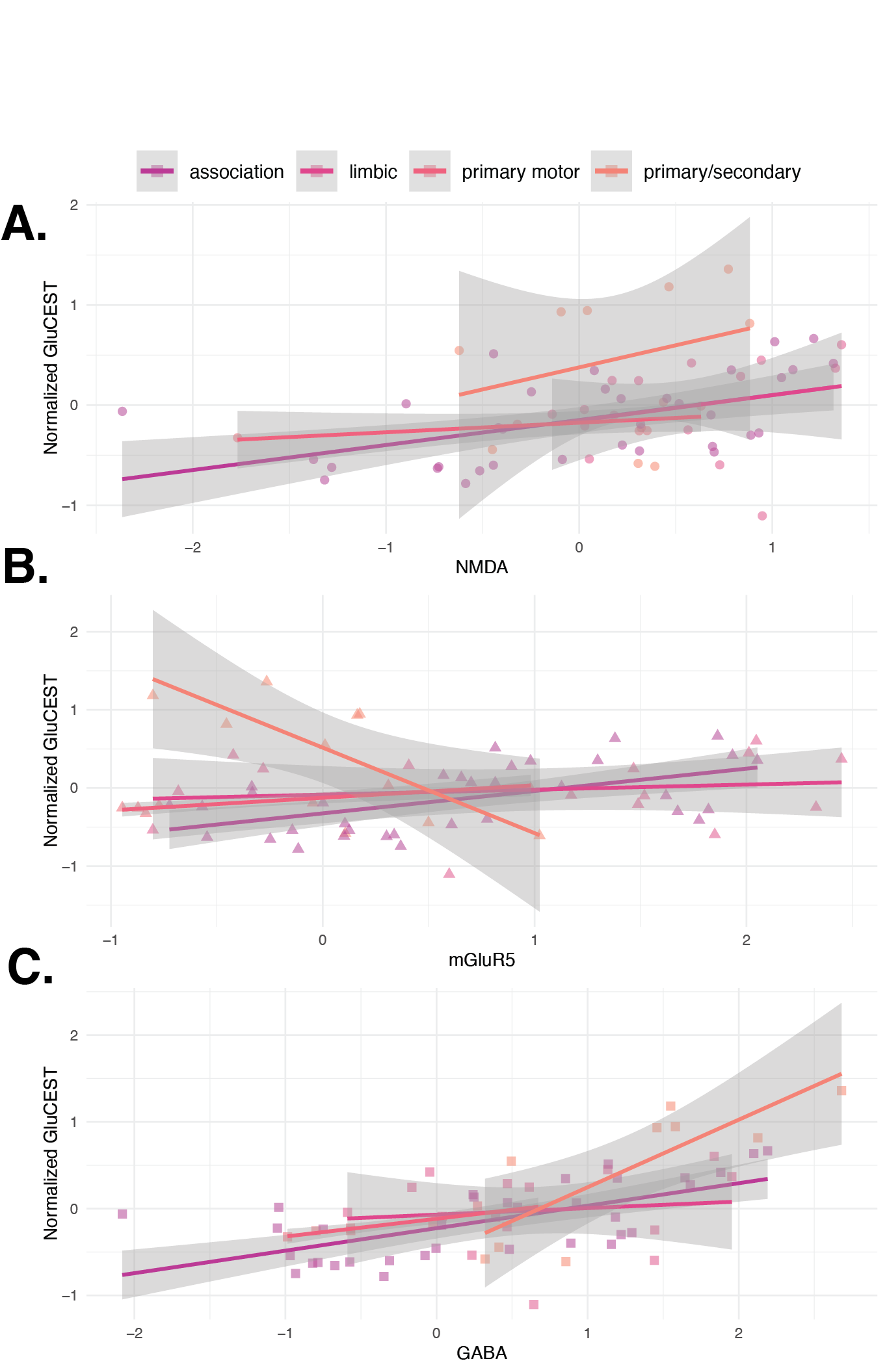


**Figure S4:** **GluCEST-Receptor trends across von Economo regions.** There was no significant interaction between NMDA and von Economo region (F(3,57) = 0.28, p_FDR_ > 0.05). However, there was an interaction between von Economo region and both mGluR5 (F(3,57) = 8.1, p_FDR_ < 0.001) and GABA_A_ (F(3,57) = 2.7, p_FDR_ = 0.08) in their respective models.

**Figure S5:** **GluCEST-Receptor trends across von Economo regions in the replication dataset.** There was no significant interaction between NMDA and von Economo region (F(3,57) = 0.6, p_FDR_ > 0.05). However, there was an interaction between von Economo region and both mGluR5 (F(3,57) = 12.7, p_FDR_ < 0.001) and GABA_A_ (F(3,57) = 5.6, p_FDR_ = 0.003) in their respective models.

**Regional interactions at various parcel inclusion thresholds**

20% parcel inclusion threshold:

- There was a significant interaction between von Economo region and NMDA (F(4,87) = 4.3, p < 0.005) and mGluR5 (F(4,87) = 15.3, p < 0.0001), but no significant interaction for GABA_A_ (F(4,87) = 0.18, p = 0.9).

40% parcel inclusion threshold:

- There was no significant interaction between von Economo region and NMDA (F(4,74) = 1.2, p =0.3) or GABA_A_ (F(4,74) = 1.14, p = 0.4), but there was a significant interaction for mGluR5 (F(4,74) = 13.8, p < 0.001).

80% parcel inclusion threshold:

- All parcels from the primary/secondary sensory cortex were excluded at this threshold. Among remaining parcels in the association, limbic, and primary motor cortices, there was no significant interaction for NMDA or mGluR5. However, there was a significant interaction for GABA_A_ (F(4,74) = 4.1, p = 0.03)


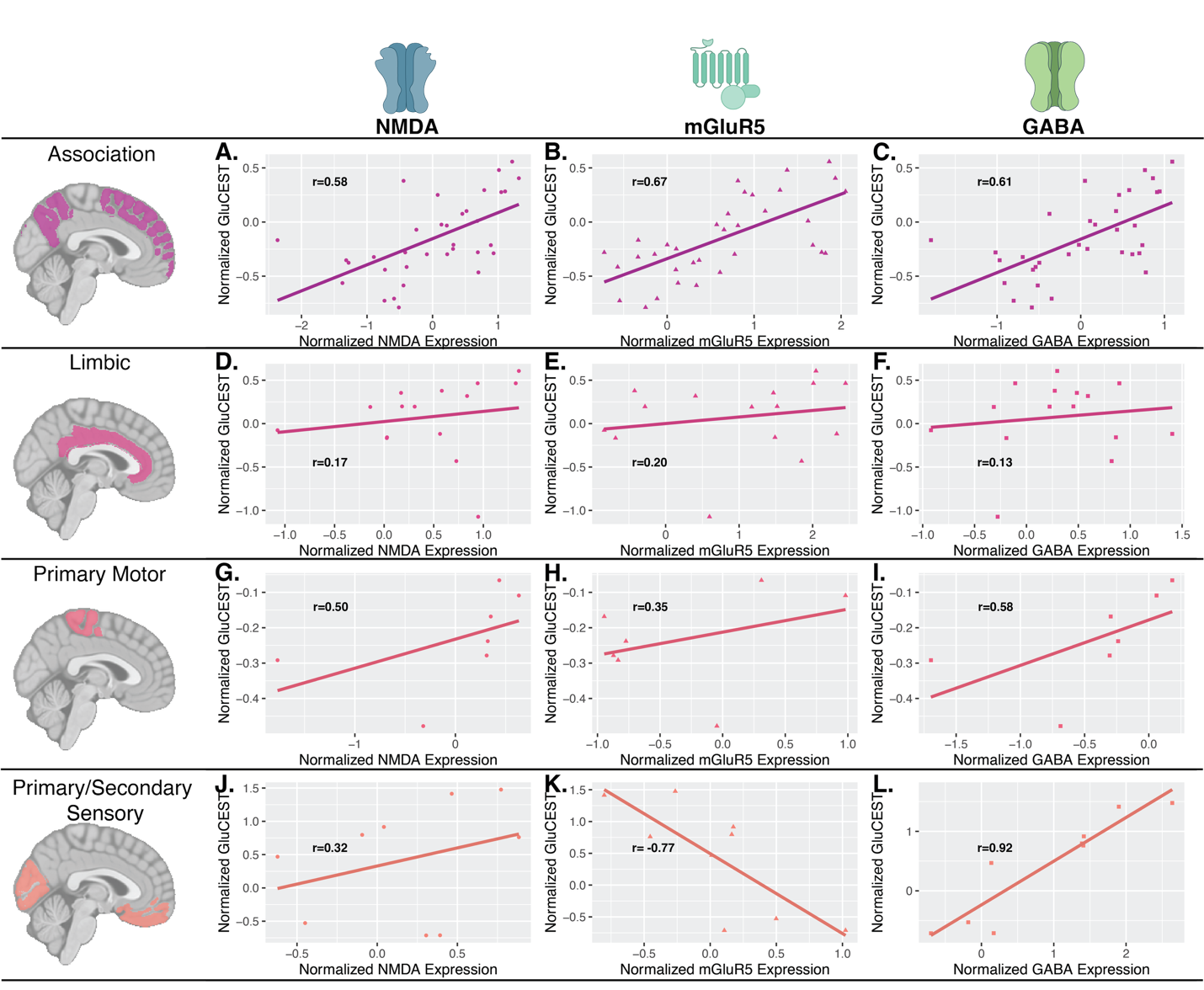


**Figure S6: GluCEST-receptor trends in each von Economo region for reliability dataset**

Results are shown for the replication dataset. Each plot shows the relationship between normalized GluCEST contrast and receptor density (as measured by normative PET maps) within different cortical zones. For NMDA, mGluR5, and GABA_A_, a positive GluCEST-receptor association was found in the association cortex (**A-C)**, limbic cortex (**D-F**), and primary motor cortex (**G-I**). In the primary/secondary sensory cortex (**J-L**), GluCEST showed a positive association with GABA_A_ and NMDA and a negative association with mGluR5.

**GluCEST-receptor trends in each von Economo region correcting for age, sex, and diagnostic status**

- Results were consistent with the main analysis. Positive GluCEST-receptor trends were identified across all regions for each receptor, with the exception of the GluCEST-mGluR5 trend in the primary/secondary sensory cortex, which was negative (r=-0.74).

**GluCEST-receptor trends in each von Economo region at various parcel inclusion thresholds**

20% parcel inclusion threshold:

- Most trends were consistent with the main analysis, with predominantly positive GluCEST-receptor associations in the association, limbic, and primary motor cortices. A negative GluCEST-NMDA association was found in the association cortex (r=-0.09) and primary/secondary sensory cortex (r=-0.5).

40% parcel inclusion threshold:

- Most trends were consistent with the main analysis, with predominantly positive GluCEST-receptor associations in the association, limbic, and primary motor cortices. A negative GluCEST-NMDA association was found in the association cortex (r=-0.1) and primary/secondary sensory cortex (r=-0.5).

80% parcel inclusion threshold:

- All parcels from the primary/secondary sensory cortex were excluded at this threshold. Among remaining parcels in the association and primary motor cortices, all trends were positive, consistent with the main analysis. In the limbic cortex, a there was a positive GluCEST-NMDA trend (r=0.52) but a negative trend for GluCEST-mGluR5 (r=-0.22) and GluCEST-GABA_A_ (r=-0.82).

**GluCEST-gene trends in main dataset prior to FDR-correction**

- GRIN1: r = 0.001, p_spin,no FDR_ = 0.95
- GRIN2A: r = 0.33, p_spin,no FDR_ = 0.056
- GRM5: r = -0.007, p_spin,no FDR_ = 0.97
- GABRA1: r = 0.21, p_spin,no FDR_ = 0.21
- GABRG2: r = 0.21, p_spin,no FDR_ = 0.30
- GLS: r = 0.34, p_spin,no FDR_ = 0.004
- GLUL: r = -0.25, p_spin,no FDR_ = 0.075

**
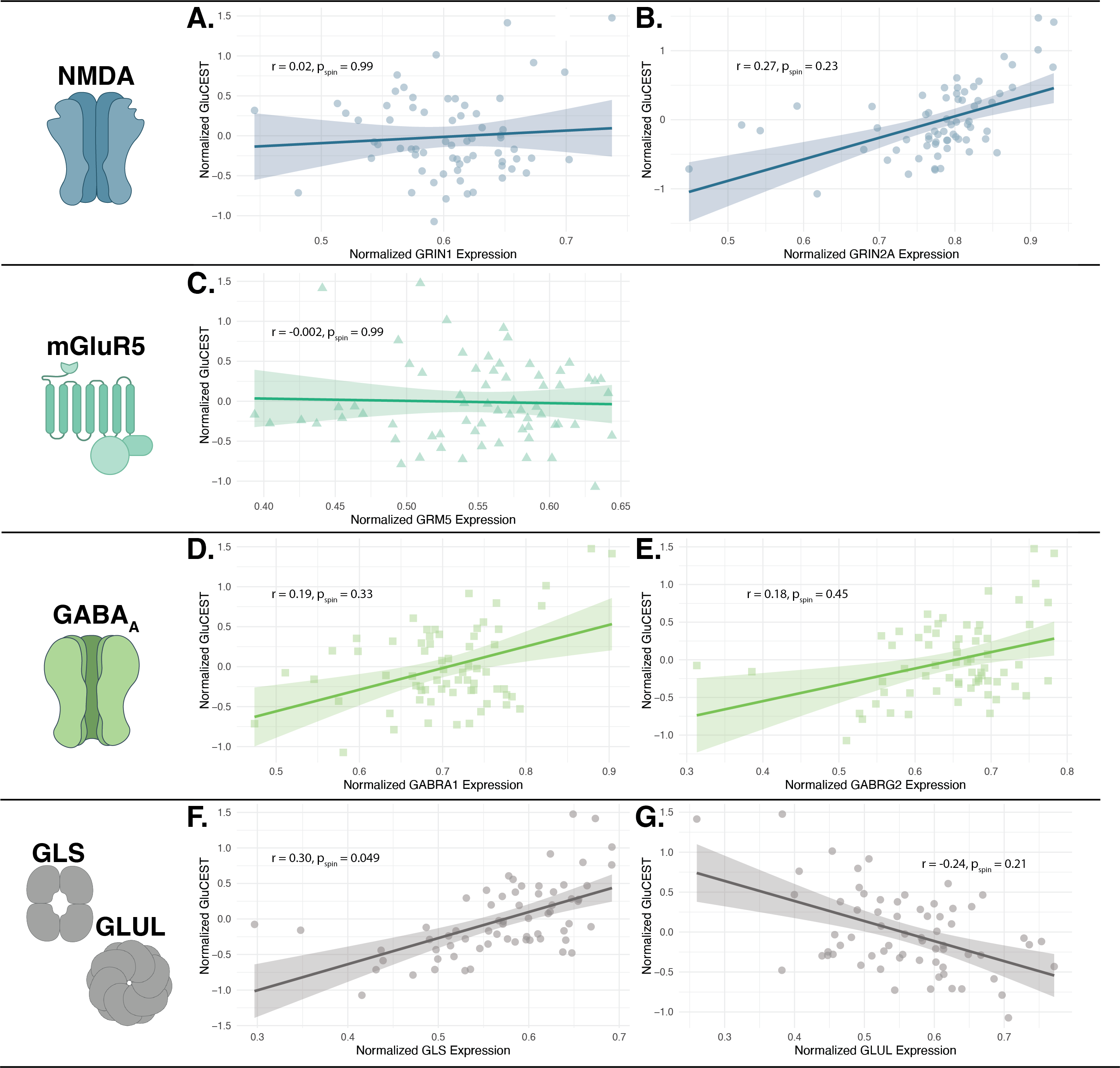
**

**Figure S7: GluCEST-gene trends in replication dataset.**

In the replication dataset (n = 53), there was a positive association between GluCEST and **GLS** (r = 0.30, p_spin_ = 0.049). There were no significant associations with **GRIN1** (r = 0.02, p_spin_ = 0.99), **GRIN2A** (r = 0.27, p_spin_ = 0.23), **GRM5** (r = -0.002, p_spin_ = 0.99), **GABRA1** (r = 0.19, p_spin_ = 0.33), **GABRG2** (r = 0.18, p_spin_ = 0.45), or **GLUL** (r = -0.24, p_spin_ = 0.21). These results are consistent with our primary exploratory analysis.

**GluCEST-gene trends in replication dataset prior to FDR-correction:**

- GRIN1: r = 0.02, p_spin,no FDR_ = 0.87
- GRIN2A: r = 0.27, p_spin,no FDR_ = 0.10
- GRM5: r = -0.002, p_spin,no FDR_ = 0.99
- GABRA1: r = 0.19, p_spin,no FDR_ = 0.19
- GABRG2: r = 0.18, p_spin,no FDR_ = 0.32
- GLS: r = 0.30, p_spin,no FDR_ = 0.007
- GLUL: r = -0.24, p_spin,no FDR_ = 0.06

**GluCEST-gene trends corrected for potential confounding variables.**

To evaluate the robustness of parcel-wise correlations between GluCEST and gene expression, we conducted several sensitivity analyses using residualized GluCEST data. Specifically, we corrected for the following covariates in separate models:

- Age, sex, and diagnostic group
- Age, sex, and individual SIPS subscale scores (Positive, Negative, Disorganized)
- Age, sex, diagnosis, and motion parameters (mean and maximum)

Across all models, the spatial correlation results remained consistent with the primary analysis.

**GluCEST-gene expression trends at various parcel inclusion thresholds (all FDR-corrected):**

20% Parcel Inclusion Threshold:

- GRIN1: r = 0.10, p_spin_ = 0.52
- GRIN2A: r = 0.35, p_spin_ = 0.09
- GRM5: r = -0.019, p_spin_ = 0.94
- GABRA1: r = 0.26, p_spin_ = 0.13
- GABRG2: r = 0.24, p_spin_ = 0.27
- GLS: r = 0.34, p_spin_ = 0.028
- GLUL: r = -0.31, p_spin_ = 0.03

40% Parcel Inclusion Threshold:

- **GRIN1:** r = 0.072, p_spin_ = 0.74
- **GRIN2A:** r = 0.34, p_spin_ = 0.09
- **GRM5:** r = -0.019, p_spin_ = 0.94
- **GABRA1:** r = 0.26, p_spin_ = 0.12
- **GABRG2:** r = 0.23, p_spin_ = 0.29
- **GLS:** r = 0.33, p_spin_ = 0.02
- **GLUL:** r = -0.29, p_spin_ = 0.045

80% Parcel Inclusion Threshold:

- **GRIN1:** r = -0.18, p_spin_ = 0.31
- **GRIN2A:** r = 0.26, p_spin_ = 0.31
- **GRM5:** r = 0.076, p_spin_ = 0.84
- **GABRA1:** r = -0.080, p_spin_ = 0.84
- **GABRG2:** r = 0.043, p_spin_ = 0.84
- **GLS:** r = 0.25, p_spin_ = 0.28
- **GLUL:** r = -0.075, p_spin_ = 0.84

GluCEST-gene density trends with means-level data

- **GRIN1:** r = 0.040, p_spin_ = 0.92
- **GRIN2A:** r = 0.61, p_spin_ = 0.12
- **GRM5:** r = -0.048, p_spin_ = 0.92
- **GABRA1:** r = 0.43, p_spin_ = 0.33
- **GABRG2:** r = 0.42, p_spin_ = 0.33
- **GLS:** r = 0.65, p_spin_ = 0.02
- **GLUL:** r = -0.50, p_spin_ = 0.12

**Supplementary References**

Ametamey, S. M., Kessler, L. J., Honer, M., Wyss, M. T., Buck, A., Hintermann, S., Auberson, Y. P., Gasparini, F., & Schubiger, P. A. (2006). Radiosynthesis and preclinical evaluation of 11C-ABP688 as a probe for imaging the metabotropic glutamate receptor subtype 5. *Journal of Nuclear Medicine: Official Publication, Society of Nuclear Medicine*, *47*(4), 698–705.

Ametamey, S. M., Treyer, V., Streffer, J., Wyss, M. T., Schmidt, M., Blagoev, M., Hintermann, S., Auberson, Y., Gasparini, F., Fischer, U. C., & Buck, A. (2007). Human PET studies of metabotropic glutamate receptor subtype 5 with 11C-ABP688. *Journal of Nuclear Medicine: Official Publication, Society of Nuclear Medicine*, *48*(2), 247–252.

Andersen, J. V., & Schousboe, A. (2023). Glial Glutamine Homeostasis in Health and Disease. *Neurochemical Research*, *48*(4), 1100–1128. https://doi.org/10.1007/s11064-022-03771-1

Arnatkeviciute, A., Fulcher, B. D., & Fornito, A. (2019). A practical guide to linking brain-wide gene expression and neuroimaging data. *NeuroImage*, *189*, 353–367. https://doi.org/10.1016/j.neuroimage.2019.01.011

Beliveau, V., Ganz, M., Feng, L., Ozenne, B., Højgaard, L., Fisher, P. M., Svarer, C., Greve, D. N., & Knudsen, G. M. (2017). A High-Resolution In Vivo Atlas of the Human Brain’s Serotonin System. *The Journal of Neuroscience: The Official Journal of the Society for Neuroscience*, *37*(1), 120–128. https://doi.org/10.1523/JNEUROSCI.2830-16.2016

Cardona, C., Sánchez-Mejías, E., Dávila, J. C., Martín-Rufián, M., Campos-Sandoval, J. A., Vitorica, J., Alonso, F. J., Matés, J. M., Segura, J. A., Norenberg, M. D., Rama Rao, K. V., Jayakumar, A. R., Gutiérrez, A., & Márquez, J. (2015). Expression of Gls and Gls2 glutaminase isoforms in astrocytes. *Glia*, *63*(3), 365–382. https://doi.org/10.1002/glia.22758

Chou, T.-H., Kang, H., Simorowski, N., Traynelis, S. F., & Furukawa, H. (2022). Structural insights into assembly and function of GluN1-2C, GluN1-2A-2C, and GluN1-2D NMDARs. *Molecular Cell*, *82*(23), 4548-4563.e4. https://doi.org/10.1016/j.molcel.2022.10.008

Corti, C., Clarkson, R. W. E., Crepaldi, L., Sala, C. F., Xuereb, J. H., & Ferraguti, F. (2003). Gene structure of the human metabotropic glutamate receptor 5 and functional analysis of its multiple promoters in neuroblastoma and astroglioma cells. *The Journal of Biological Chemistry*, *278*(35), 33105–33119. https://doi.org/10.1074/jbc.M212380200

Daggett, L. P., Sacaan, A. I., Akong, M., Rao, S. P., Hess, S. D., Liaw, C., Urrutia, A., Jachec, C., Ellis, S. B., & Dreessen, J. (1995). Molecular and functional characterization of recombinant human metabotropic glutamate receptor subtype 5. *Neuropharmacology*, *34*(8), 871–886. https://doi.org/10.1016/0028-3908(95)00085-k

DuBois, J. M., Rousset, O. G., Rowley, J., Porras-Betancourt, M., Reader, A. J., Labbe, A., Massarweh, G., Soucy, J.-P., Rosa-Neto, P., & Kobayashi, E. (2016). Characterization of age/sex and the regional distribution of mGluR5 availability in the healthy human brain measured by high-resolution [11C]ABP688 PET. *European Journal of Nuclear Medicine and Molecular Imaging*, *43*(1), 152–162. https://doi.org/10.1007/s00259-015-3167-6

Dukart, J., Holiga, Š., Chatham, C., Hawkins, P., Forsyth, A., McMillan, R., Myers, J., Lingford-Hughes, A. R., Nutt, D. J., Merlo-Pich, E., Risterucci, C., Boak, L., Umbricht, D., Schobel, S., Liu, T., Mehta, M. A., Zelaya, F. O., Williams, S. C., Brown, G., … Sambataro, F. (2018). Cerebral blood flow predicts differential neurotransmitter activity. *Scientific Reports*, *8*(1), 4074. https://doi.org/10.1038/s41598-018-22444-0

Ettrup, A., da Cunha-Bang, S., McMahon, B., Lehel, S., Dyssegaard, A., Skibsted, A. W., Jørgensen, L. M., Hansen, M., Baandrup, A. O., Bache, S., Svarer, C., Kristensen, J. L., Gillings, N., Madsen, J., & Knudsen, G. M. (2014). Serotonin 2A receptor agonist binding in the human brain with [^11^C]Cimbi-36. *Journal of Cerebral Blood Flow and Metabolism: Official Journal of the International Society of Cerebral Blood Flow and Metabolism*, *34*(7), 1188–1196. https://doi.org/10.1038/jcbfm.2014.68

Frankle, W. G., Cho, R. Y., Prasad, K. M., Mason, N. S., Paris, J., Himes, M. L., Walker, C., Lewis, D. A., & Narendran, R. (2015). In vivo measurement of GABA transmission in healthy subjects and schizophrenia patients. *The American Journal of Psychiatry*, *172*(11), 1148–1159. https://doi.org/10.1176/appi.ajp.2015.14081031

Fulcher, B. D., Little, M. A., & Jones, N. S. (2013). Highly comparative time-series analysis: The empirical structure of time series and their methods. *Journal of The Royal Society Interface*, *10*(83), 20130048. https://doi.org/10.1098/rsif.2013.0048

Galovic, M., Erlandsson, K., Fryer, T. D., Hong, Y. T., Manavaki, R., Sari, H., Chetcuti, S., Thomas, B. A., Fisher, M., Sephton, S., Canales, R., Russell, J. J., Sander, K., Årstad, E., Aigbirhio, F. I., Groves, A. M., Duncan, J. S., Thielemans, K., Hutton, B. F., … NEST investigators. (2021). Validation of a combined image derived input function and venous sampling approach for the quantification of [18F]GE-179 PET binding in the brain. *NeuroImage*, *237*, 118194. https://doi.org/10.1016/j.neuroimage.2021.118194

Hansen, J. Y., Shafiei, G., Markello, R. D., Smart, K., Cox, S. M. L., Norgaard, M., Beliveau, V., Wu, Y., Gallezot, J. D., Aumont, E., Servaes, S., Scala, S. G., DuBois, J. M., Wainstein, G., Bezgin, G., Funck, T., Schmitz, T. W., Spreng, R. N., Galovic, M., … Misic, B. (2022). Mapping neurotransmitter systems to the structural and functional organization of the human neocortex. *Nat Neurosci*, *25*(11), 1569–1581. https://doi.org/10.1038/s41593-022-01186-3

Hawrylycz, M., Miller, J. A., Menon, V., Feng, D., Dolbeare, T., Guillozet-Bongaarts, A. L., Jegga, A. G., Aronow, B. J., Lee, C.-K., Bernard, A., Glasser, M. F., Dierker, D. L., Menche, J., Szafer, A., Collman, F., Grange, P., Berman, K. A., Mihalas, S., Yao, Z., … Lein, E. (2015). Canonical genetic signatures of the adult human brain. *Nature Neuroscience*, *18*(12), 1832–1844. https://doi.org/10.1038/nn.4171

McGinnity, C. J., Hammers, A., Barros, D. A. R., Luthra, S. K., Jones, P. A., Trigg, W., Micallef, C., Symms, M. R., Brooks, D. J., Koepp, M. J., & Duncan, J. S. (2014). Initial Evaluation of 18F-GE-179, a Putative PET Tracer for Activated N-Methyl d-Aspartate Receptors. *Journal of Nuclear Medicine*, *55*(3), 423–430. https://doi.org/10.2967/jnumed.113.130641

Nørgaard, M., Beliveau, V., Ganz, M., Svarer, C., Pinborg, L. H., Keller, S. H., Jensen, P. S., Greve, D. N., & Knudsen, G. M. (2021). A high-resolution in vivo atlas of the human brain’s benzodiazepine binding site of GABAA receptors. *NeuroImage*, *232*, 117878. https://doi.org/10.1016/j.neuroimage.2021.117878

Quackenbush, J. (2002). Microarray data normalization and transformation. *Nature Genetics*, *32 Suppl*, 496–501. https://doi.org/10.1038/ng1032

Riss, P. J., Hong, Y. T., Williamson, D., Caprioli, D., Sitnikov, S., Ferrari, V., Sawiak, S. J., Baron, J.-C., Dalley, J. W., Fryer, T. D., & Aigbirhio, F. I. (2011). Validation and quantification of [18F]altanserin binding in the rat brain using blood input and reference tissue modeling. *Journal of Cerebral Blood Flow and Metabolism: Official Journal of the International Society of Cerebral Blood Flow and Metabolism*, *31*(12), 2334–2342. https://doi.org/10.1038/jcbfm.2011.94

Romero-Garcia, R., Whitaker, K. J., Váša, F., Seidlitz, J., Shinn, M., Fonagy, P., Dolan, R. J., Jones, P. B., Goodyer, I. M., Bullmore, E. T., & Vértes, P. E. (2018). Structural covariance networks are coupled to expression of genes enriched in supragranular layers of the human cortex. *NeuroImage*, *171*, 256–267. https://doi.org/10.1016/j.neuroimage.2017.12.060

Rose, C. F., Verkhratsky, A., & Parpura, V. (2013). Astrocyte glutamine synthetase: Pivotal in health and disease. *Biochemical Society Transactions*, *41*(6), 1518–1524. https://doi.org/10.1042/BST20130237

Savli, M., Bauer, A., Mitterhauser, M., Ding, Y.-S., Hahn, A., Kroll, T., Neumeister, A., Haeusler, D., Ungersboeck, J., Henry, S., Isfahani, S. A., Rattay, F., Wadsak, W., Kasper, S., & Lanzenberger, R. (2012). Normative database of the serotonergic system in healthy subjects using multi-tracer PET. *NeuroImage*, *63*(1), 447–459. https://doi.org/10.1016/j.neuroimage.2012.07.001

Sigel, E., & Steinmann, M. E. (2012). Structure, function, and modulation of GABA(A) receptors. *The Journal of Biological Chemistry*, *287*(48), 40224–40231. https://doi.org/10.1074/jbc.R112.386664

Smart, K., Cox, S. M. L., Scala, S. G., Tippler, M., Jaworska, N., Boivin, M., Séguin, J. R., Benkelfat, C., & Leyton, M. (2019). Sex differences in [11C]ABP688 binding: A positron emission tomography study of mGlu5 receptors. *European Journal of Nuclear Medicine and Molecular Imaging*, *46*(5), 1179–1183. https://doi.org/10.1007/s00259-018-4252-4

Song, R., Zhang, J., Perszyk, R. E., Camp, C. R., Tang, W., Kannan, V., Li, J., Xu, Y., Chen, J., Li, Y., Liang, S. H., Traynelis, S. F., & Yuan, H. (2024). Differential responses of disease-related GRIN variants located in pore-forming M2 domain of N-methyl-D-aspartate receptor to FDA-approved inhibitors. *Journal of Neurochemistry*, *168*(12), 3936–3949. https://doi.org/10.1111/jnc.15942

Stojanovic, T., Capo, I., Aronica, E., Adle-Biassette, H., Höger, H., Sieghart, W., Kovacs, G. G., & Milenkovic, I. (2016). The α1, α2, α3, and γ2 subunits of GABAA receptors show characteristic spatial and temporal expression patterns in rhombencephalic structures during normal human brain development. *The Journal of Comparative Neurology*, *524*(9), 1805–1824. https://doi.org/10.1002/cne.23923

Sun, Y., Cheng, X., Zhang, L., Hu, J., Chen, Y., Zhan, L., & Gao, Z. (2017). The Functional and Molecular Properties, Physiological Functions, and Pathophysiological Roles of GluN2A in the Central Nervous System. *Molecular Neurobiology*, *54*(2), 1008–1021. https://doi.org/10.1007/s12035-016-9715-7

Syvänen, S., de Lange, E. C., Tagawa, Y., Schenke, M., Molthoff, C. F. M., Windhorst, A. D., Lammertsma, A. A., & Voskuyl, R. A. (2011). Simultaneous in vivo measurements of receptor density and affinity using [11C]flumazenil and positron emission tomography: Comparison of full saturation and steady state methods. *NeuroImage*, *57*(3), 928–937. https://doi.org/10.1016/j.neuroimage.2011.05.022

Tecson, M. C. B., Geluz, C., Cruz, Y., & Greene, E. R. (2025). Glutamine Synthetase: Diverse Regulation and Functions of an Ancient Enzyme. *Biochemistry*, *64*(3), 547–554. https://doi.org/10.1021/acs.biochem.4c00763

van Kuilenburg, A. B. P., Tarailo-Graovac, M., Richmond, P. A., Drögemöller, B. I., Pouladi, M. A., Leen, R., Brand-Arzamendi, K., Dobritzsch, D., Dolzhenko, E., Eberle, M. A., Hayward, B., Jones, M. J., Karbassi, F., Kobor, M. S., Koster, J., Kumari, D., Li, M., MacIsaac, J., McDonald, C., … van Karnebeek, C. D. M. (2019). Glutaminase Deficiency Caused by Short Tandem Repeat Expansion in GLS. *The New England Journal of Medicine*, *380*(15), 1433–1441. https://doi.org/10.1056/NEJMoa1806627

Vibholm, A. K., Dietz, M. J., Beniczky, S., Christensen, J., Højlund, A., Jacobsen, J., Bender, D., Møller, A., & Brooks, D. J. (2021). Activated N-methyl-D-aspartate receptor ion channels detected in focal epilepsy with [18 F]GE-179 positron emission tomography. *Epilepsia*, *62*(12), 2899–2908. https://doi.org/10.1111/epi.17074

Yashiro, K., & Philpot, B. D. (2008). Regulation of NMDA Receptor Subunit Expression and Its Implications for LTD, LTP, and Metaplasticity. *Neuropharmacology*, *55*(7), 1081–1094. https://doi.org/10.1016/j.neuropharm.2008.07.046

Zhu, S., Noviello, C. M., Teng, J., Walsh, R. M., Kim, J. J., & Hibbs, R. E. (2018). Structure of a human synaptic GABAA receptor. *Nature*, *559*(7712), 67–72. https://doi.org/10.1038/s41586-018-0255-3
